# Inferring Influenza Infection Attack Rate from Seroprevalence Data

**DOI:** 10.1371/journal.ppat.1004054

**Published:** 2014-04-03

**Authors:** Joseph T. Wu, Kathy Leung, Ranawaka A. P. M. Perera, Daniel K. W. Chu, Cheuk Kwong Lee, Ivan F. N. Hung, Che Kit Lin, Su-Vui Lo, Yu-Lung Lau, Gabriel M. Leung, Benjamin J. Cowling, J. S. Malik Peiris

**Affiliations:** 1 Department of Community Medicine and School of Public Health, Li Ka Shing Faculty of Medicine, The University of Hong Kong, Hong Kong Special Administrative Region, People's Republic of China; 2 Centre of Influenza Research and School of Public Health, Li Ka Shing Faculty of Medicine, The University of Hong Kong, Hong Kong Special Administrative Region, People's Republic of China; 3 Hong Kong Red Cross Blood Transfusion Service, Hospital Authority, Hong Kong Special Administrative Region, People's Republic of China; 4 Department of Medicine, Li Ka Shing Faculty of Medicine, The University of Hong Kong, Hong Kong Special Administrative Region, People's Republic of China; 5 Hospital Authority, Hong Kong Special Administrative Region, People's Republic of China; 6 Food and Health Bureau, Government of the Hong Kong Special Administrative Region, Hong Kong Special Administrative Region, People's Republic of China; 7 Department of Paediatrics and Adolescent Medicine, Li Ka Shing Faculty of Medicine, The University of Hong Kong, Hong Kong Special Administrative Region, People's Republic of China; 8 HKU-Pasteur Research Pole, Centre of Influenza Research and School of Public Health, Li Ka Shing Faculty of Medicine, The University of Hong Kong, Hong Kong Special Administrative Region, People's Republic of China; Imperial College London, United Kingdom

## Abstract

Seroprevalence survey is the most practical method for accurately estimating infection attack rate (IAR) in an epidemic such as influenza. These studies typically entail selecting an arbitrary titer threshold for seropositivity (e.g. microneutralization [MN] 1∶40) and assuming the probability of seropositivity given infection (infection-seropositivity probability, ISP) is 100% or similar to that among clinical cases. We hypothesize that such conventions are not necessarily robust because different thresholds may result in different IAR estimates and serologic responses of clinical cases may not be representative. To illustrate our hypothesis, we used an age-structured transmission model to fully characterize the transmission dynamics and seroprevalence rises of 2009 influenza pandemic A/H1N1 (pdmH1N1) during its first wave in Hong Kong. We estimated that while 99% of pdmH1N1 infections became MN_1∶20_ seropositive, only 72%, 62%, 58% and 34% of infections among age 3–12, 13–19, 20–29, 30–59 became MN_1∶40_ seropositive, which was much lower than the 90%–100% observed among clinical cases. The fitted model was consistent with prevailing consensus on pdmH1N1 transmission characteristics (e.g. initial reproductive number of 1.28 and mean generation time of 2.4 days which were within the consensus range), hence our ISP estimates were consistent with the transmission dynamics and temporal buildup of population-level immunity. IAR estimates in influenza seroprevalence studies are sensitive to seropositivity thresholds and ISP adjustments which in current practice are mostly chosen based on conventions instead of systematic criteria. Our results thus highlighted the need for reexamining conventional practice to develop standards for analyzing influenza serologic data (e.g. real-time assessment of bias in ISP adjustments by evaluating the consistency of IAR across multiple thresholds and with mixture models), especially in the context of pandemics when robustness and comparability of IAR estimates are most needed for informing situational awareness and risk assessment. The same principles are broadly applicable for seroprevalence studies of other infectious disease outbreaks.

## Author Summary

Seroprevalence studies have been regarded as the most practical method for accurately estimating the number of infections in influenza epidemics and pandemics. However, methods for inferring the number of infections from seroprevalence data in previous studies have mostly been based on conventional practice instead of standardized criteria. Specifically, there are no systematic criteria on how to select the seropositivity threshold and adjust for the proportion of infections that become seropositive. Here, we showed that under the conventional criteria, the number of 2009 pandemic influenza A/H1N1 infections had been substantially underestimated in Hong Kong as well as other countries, mostly due to overestimation of the proportion of infections that became seropositive. Our results highlighted the need to reexamine the widely accepted practice in interpreting seroprevalence data, especially in the context of pandemics when little is known but robust and comparable estimates of the number of infections and severity are most needed for informing situational awareness and guiding control policies.

## Introduction

Severity of influenza infection is defined as the probability of severe complications (e.g. hospitalization or death) if infected [1]. Timely and accurate estimates of severity are extremely valuable for informing decisions about the scale and targeting of response to an emerging pandemic [2]. In 2011, the International Health Regulations Review Committee highlighted the lack of “a consistent, measurable and understandable depiction of severity” as a major shortcoming of global response to the 2009 influenza pandemic [3]. Real-time serial cross-sectional or longitudinal seroprevalence studies can address this shortcoming in future pandemics by providing direct estimates of infection attack rate (IAR) as the denominator for severity [4].

In serial cross-sectional seroprevalence studies, with the absence of vaccination, IARs are estimated from seroprevalence rise (Δ*S*). These studies typically entail selecting an arbitrary titer threshold for seropositivity. Although many influenza seroprevalence studies have been conducted, there is no consensus on how to select seropositivity thresholds and adjust for the proportion of infections that became seropositive (infection-seropositivity probability, ISP). Haemagglutinin-inhibition (HI) titer 1∶40 and microneutralization (MN) titer 1∶40 have been commonly used as seropositivity thresholds [5]; ISP has either been ignored (IAR≈Δ*S*, e.g. [6]–[11]) or assumed to be similar to the proportion of clinical cases that became seropositive during convalescence (IAR≈Δ*S*/(proportion of clinical cases seropositive), e.g. [12]–[15]). Historically, seropositivity thresholds were often chosen by conventions instead of systematic evaluation and ISP was rarely included or discussed [4]. Previous studies have noted the arbitrariness associated with predefined seropositivity thresholds and proposed to circumvent such arbitrariness by fitting the cross-sectional titer distribution to a mixture of probability distributions for estimating IAR [16]. A simple example of these so-called mixture models is the superposition of two lognormal distributions which correspond to the titer distributions of the uninfected and infected populations [17]. In this study, we incorporated such mixture model structure into a transmission model to show that conventional seropositivity thresholds and ISP adjustments had probably led to underestimation of IARs in many seroprevalence studies of 2009 pandemic influenza A/H1N1 (pdmH1N1). Our results thus resonate with these earlier studies regarding the lack of robustness in conventional practice for inferring IAR from seroprevalence data, not only for influenza but also other infectious diseases [18], [19]. Our results highlighted the need to reexamine the widely accepted practice in interpreting seroprevalence data, especially in the context of pandemics when little is known but robust and comparable estimates of the number of infections and severity are most needed for informing situational awareness and guiding control policies.

## Results

### Seroprevalence data

**Figure 1:**
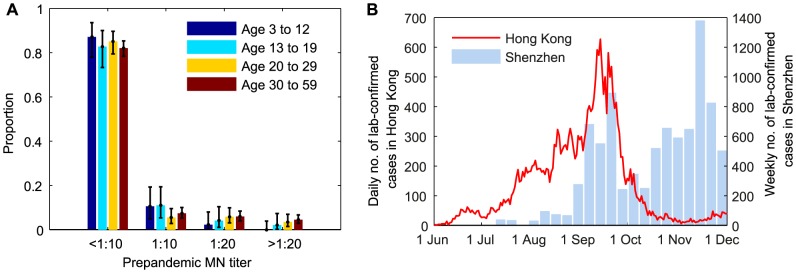
Prepandemic seroprevalence and the epidemic curve of pdmH1N1 in Hong Kong. **A** Age-stratified pre-pandemic MN titer distributions which were estimated from serum samples collected in June and early-July 2009. For samples collected after July 2009, we only tested whether they were MN_1∶20_ and MN_1:40_ seropositive because of logistical constraints. **B** Epidemic curves of pdmH1N1 in Hong Kong and Shenzhen. Estimated weekly numbers of lab-confirmed cases in Shenzhen were extracted from [38].

During the 2009 influenza pandemic in Hong Kong, we conducted a large serial cross-sectional seroprevalence study with ∼14,800 serum samples from individuals aged 3–59 years, the details of which have been previously documented [11], [13]. Briefly, for samples collected before or in July 2009, we tested whether they were seropositive with respect to MN titer 1∶10, 1∶20, 1∶40, 1∶80, 1∶160, 1∶320, 1∶640, 1∶1280, and 1∶2560 (Figure 1A). Due to logistical constraints, for samples collected after July 2009, we only tested whether they were MN_1∶20_ and MN_1∶40_ seropositive, e.g. if a sample was MN_1∶80_ seropositive, we would only know that it was MN_1∶20_ and MN_1∶40_ seropositive. We denoted the seroprevalence, seroprevalence rise and infection-seropositivity probability for MN_1:*X*_ by *S_X_*, Δ*S_X_* and *ISP_X_*, respectively.

### Hospitalization data

The bulk of the first wave of pdmH1N1 in Hong Kong occurred between 1 June and 30 November 2009 (Figure 1B). Age-stratified daily number of pdmH1N1 hospitalizations during this period was provided by the Hong Kong Hospital Authority [20], [21]. Since May 2009, patients admitted with acute respiratory illnesses routinely underwent laboratory testing for pdmH1N1 [22]. Due to containment efforts enforced until June 29, all lab-confirmed pdmH1N1 cases before that date were hospitalized for isolation regardless of their clinical conditions. Therefore, our analysis only used hospitalization data from June 30 onwards during which only those required hospital care were admitted.

### Preliminary analysis

**Figure 2:**
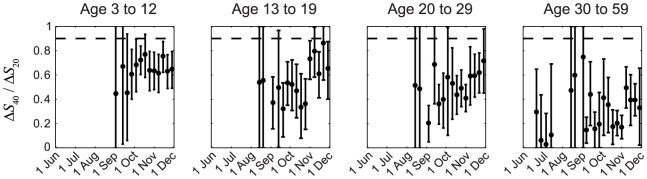
Age-specific Δ*S*_40_/Δ*S*_20_ during the first wave of pdmH1N1 in Hong Kong. Δ*S*_40_ and Δ*S*_20_ at each cross-section were estimated using the method described in our previous work [11]. If ISP_20_ and ISP_40_ (among all pdmH1N1 infections) were the same as the proportions of clinical cases that became MN_1:20_ and MN_1∶40_ seropositive (i.e. around 100% and 90%, respectively [23], [24]), Δ*S*_40_/Δ*S*_20_ should have remained close to 0.9–1 (the horizontal dashed line) throughout the first wave, which was not the case in reality as shown here.

In our previous IAR estimates, we (i) adopted the conventional MN_1∶40_ seropositivity threshold because the proportion of pdmH1N1 clinical cases who became MN_1∶20_ and MN_1∶40_ seropositive during convalescence were ∼100% and 90%, respectively [23], [24]; and (ii) assumed that ISP of all pdmH1N1 cases (i.e. including mild and asymptomatic infections) were similar to the proportion of clinical cases that became seropositive, i.e., *ISP*_20_≈1 and *ISP*_40_≈0.9–1. Because *IAR*≈Δ*S*_X_/*ISP*_X_, it follows that Δ*S*_40_/Δ*S*_20_≈*ISP*_40_/*ISP*_20_. The assumption *ISP*_20_≈1 and *ISP*_40_≈0.9–1 thus implied Δ*S*_40_/Δ*S*_20_>0.9. However, this contradicted our serial cross-sectional seroprevalence data which suggested that Δ*S*_40_/Δ*S*_20_ was consistently much smaller than 0.9 in all cross-sections throughout the first wave for all age groups, especially among older adults (Figure 2). The contribution of seasonal influenza to Δ*S*_20_ was small because (i) <34% of influenza A isolates during the first wave were seasonal influenza (http://www.chp.gov.hk/en/epidemiology/304/518/519.html); and (ii) in a Hong Kong study of within-household influenza transmission [25], only a small percentage of subjects infected with seasonal influenza became MN_1∶20_ seropositive against pdmH1N1 (unpublished data, BJ Cowling). Thus, given that pdmH1N1 vaccination was absent during the study period, Δ*S*_20_ could only be attributed to pdmH1N1 infections. This preliminary analysis strongly suggested that a substantial proportion of pdmH1N1 infections (e.g. mild and asymptomatic infections) did not become MN_1∶40_ seropositive. To substantiate this hypothesis, we developed a mathematical model to fully characterize the transmission dynamics and seroprevalence rises of pdmH1N1 during its first wave in Hong Kong.

### Transmission dynamics and ISP estimates

We used an age-structured Susceptible-Exposed-Infected-Recovered (SEIR) model with 4 age groups (age 3–12 y, 13–19 y, 20–29 y and 30–59 y) to simulate pdmH1N1 transmission between 1 June and 30 November 2009. The 0–2 and ≥60 age groups were omitted because (i) reliable serologic data from them were not available and (ii) they only represented 2% of all lab-confirmed pdmH1N1 cases and 5% of all pdmH1N1 hospitalizations and thus likely to have small contribution in pdmH1N1 transmission. In our sensitivity analysis, we showed that our results remained almost unchanged if we included these age groups in disease transmission. We used the POLYMOD matrices constructed for European countries (8 matrices and their average **P_AVG_**) as the contact matrix **C** because analogous data was unavailable from Hong Kong [26] and most of our results were insensitive to the choice of contact matrix. We included the effect of infection importations from Shenzhen, a large city adjacent to Hong Kong with a population of 13 million (Figure 1B).

**Figure 3:**
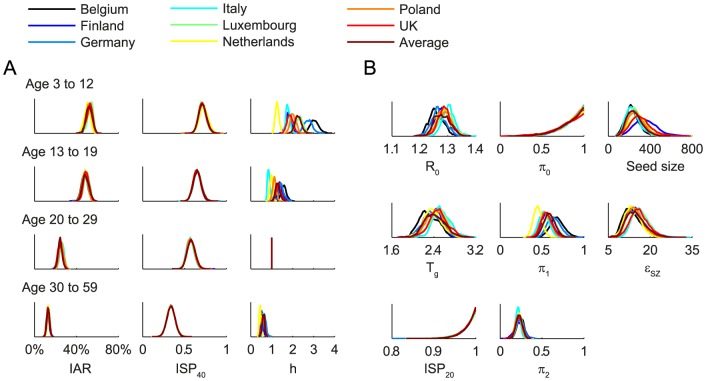
Posterior distributions of parameter estimates. Different colors correspond to different POLYMOD contact matrices. **A** Age-dependent parameters including IARs (first column), *ISP*_40_ (second), and age-specific susceptibility (third). **B** Other parameters including *R*(0), *T_g_*, *ISP*_20_, reduction in within-age-group mixing due to school closure (*π*_0_, *π*_1_, *π*_2_), seed size, and scaling factor for FOI from Shenzhen (*ε_SZ_*).

**Figure 4:**
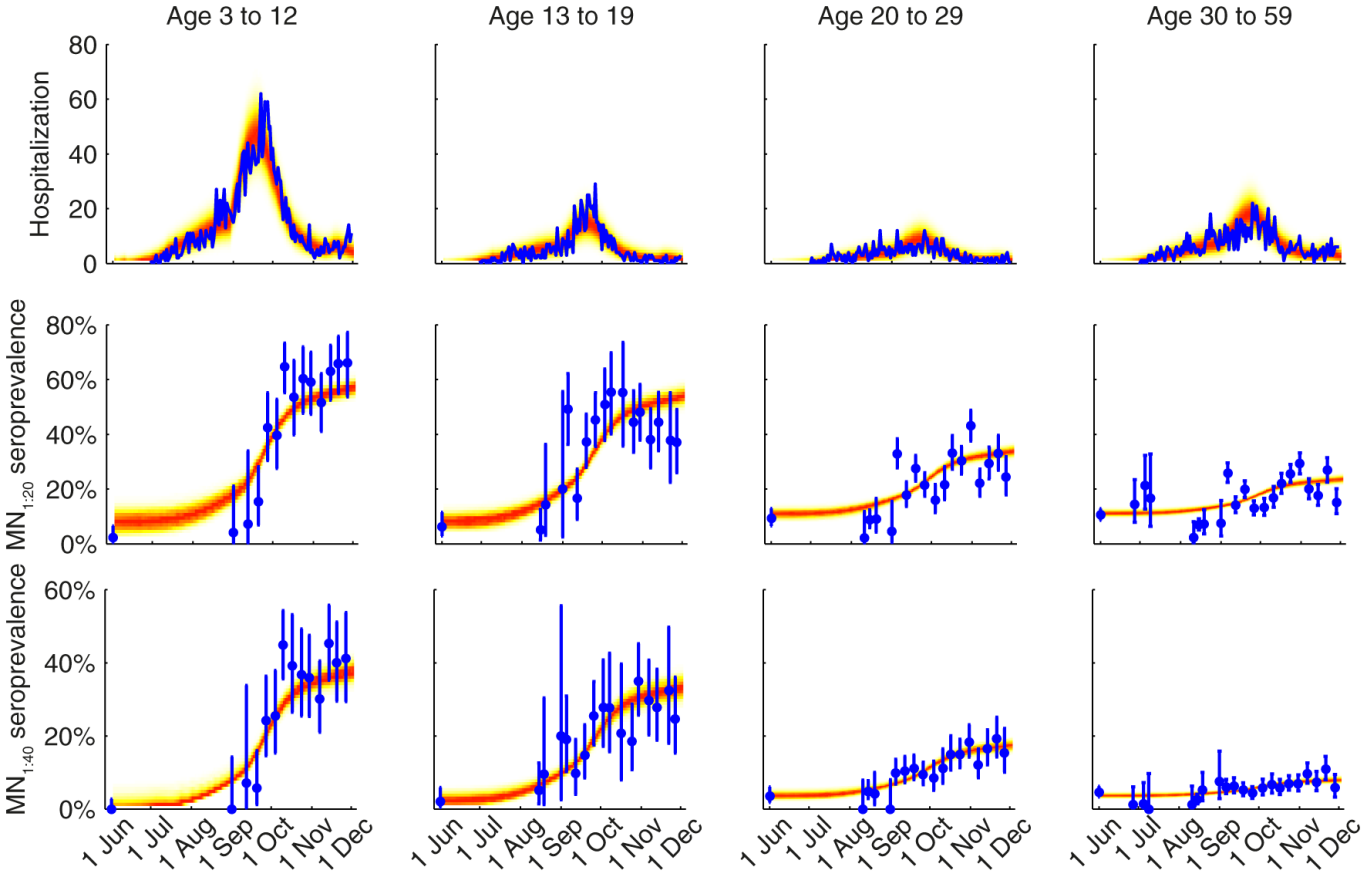
Comparison of the data and the fitted model. The hospitalization and serial cross-sectional seroprevalence data are shown in blue (vertical bars indicate 95% confidence intervals). Posterior intervals of hospitalizations and seroprevalence in the fitted model are shown as heat shades in which darker colors represent higher probability densities (i.e. highest density in red and zero density in white).

We fitted the transmission model to the seroprevalence and hospitalization data by estimating the parameters listed in Table 1. All parameters were identifiable (Figure 3 and Table 1) and the fitted model was congruent with the data (Figure 4). Parameter estimates were very similar across all nine contact matrices except for age-specific susceptibility (see below for details). Partial rank correlation coefficient (PRCC) analysis did not indicate any unexpected confounding effects (see Text S1).

**Table 1:** Model parameters and their posterior statistics.

Parameter	Description	Posterior median (95% credible interval)
*R*(0)	Initial reproductive number	1.28 (1.23–1.34)
*T_g_*	Mean generation time (days)	2.4 (2.1–2.8)
*π*_0_	Reduction in within-group transmission for the 3–12 age group during proactive school closure	86% (44%–99%)
*π*_1_, *π*_2_	Reduction in within-group transmission during summer holidays	Age 3–12: 59% (46%–73%)
		Age 13–19: 23% (15%–30%)
*x_a,i_*(0)	Proportion of age group *a* with the *i*th pre-pandemic titer level	Very similar to the distributions in Figure 1A
*h*_a_	Age-specific susceptibility of age group *a* compared to the 20–29 age group	Age 3–12: 2.3 (2–2.6)
		Age 13–19: 1.3 (1.1–1.5)
		Age 30–59: 0.6 (0.5–0.7)
*ISP*_20_	MN_1∶20_ infection-seropositivity probability	0.99 (0.93–1)
*ISP*_40,*a*_	Age-specific MN_1∶40_ infection-seropositivity probability	Age 3–12: 0.72 (0.63–0.82)
		Age 13–19: 0.65 (0.56–0.75)
		Age 20–29: 0.58 (0.49–0.68)
		Age 30–59: 0.34 (0.24–0.44)
*μ_Seropos, X_*	Mean delay (days) from onset to MN_1:*X*_ seropositivity for those infections who became MN_1:*X*_ seropositive during convalescence	MN_1∶20_: 7.3 (6.1–8.6)
		MN_1∶40_: 9.7 (7.9–11.3)
*M*	Seed size	246 (132–420)
*ε_SZ_*	Scaling factor for exogenous FOI from Shenzhen	15 (9–23)
*IHP_a_*	Age-specific infection-hospitalization probability	Age 3–12: 0.89% (0.8%–1%)
		Age 13–19: 0.29% (0.26%–0.34%)
		Age 20–29: 0.22% (0.18%–0.26%)
		Age 30–59: 0.23% (0.19%–0.29%)

We estimated that the initial reproductive number *R*(0) was 1.28 (95% credible interval, 1.23–1.34) and mean generation time *T_g_* was 2.4 (2.1–2.8) days, i.e. consistent with estimates of pdmH1N1 transmission parameters in other studies [27]. The scaling factor for the force of infection (FOI) from Shenzhen was *ε_SZ_* = 15 (9–23), which conformed with the intuition that
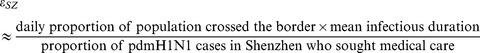
(see Text S1).

Among infected individuals who were MN_1∶20_ seronegative before infection, 99% (93%–100%) became MN_1∶20_ seropositive with a mean delay of 7.3 (6.1–8.6) days after onset. Among infected individuals who were MN_1∶40_ seronegative before infection, 72% (63%–82%), 65% (56%–75%), 58% (49%–68%) and 34% (24%–44%) among the 3–12, 13–19, 20–29 and 30–59 age group became MN_1∶40_ seropositive with a mean delay of 9.5 (7.9–11.3) days after onset. Hence, *ISP*_40_ decreased with age and was much lower compared to the 90–100% of clinical cases that became MN_1∶40_ seropositive [23], [24]. Consequently, IAR estimates here were significantly higher than our previous estimates, especially for the 30–59 age group [13]: 52% (46%–58%), 49% (43%–55%), 25% (21%–29%) and 13% (10%–16%) for age 3–12, 13–19, 20–29 and 30–59.

Proactive closure of kindergartens and primary schools reduced mixing among children aged 3–12 by 86% (44%–99%). Summer holidays reduced within-age-group mixing by 59% (46%–73%) and 23% (15%–30%) for age 3–12 and 13–19. A weaker effect of school closing for age 13–19 was plausible because older teenagers were more likely to actively mix with their peers in non-school settings while schools were closed.

Age-specific susceptibilities *h_a_*'s were sensitive to the choice of contact matrix **C** because disease transmission was essentially driven by the matrix {*W_ab_* = *h_a_C_ab_*}. For all POLYMOD matrices, adults aged 30–59 were 0.4–0.7 times as susceptible as those aged 20–29. Children aged 3–12 were ∼2–3 times as susceptible as those aged 20–29 except for the Netherlands matrix which gave an estimate of 1.3 (1.1–1.4). Children aged 13–19 were 1.1–1.6 times more susceptible than adults aged 20–29 except for the Italy matrix which gave an estimate of 0.9 (0.8–1). In summary, these age-specific susceptibility estimates were consistent with analogous estimates from studies which showed that susceptibility decreased with age after adjusting for preexisting antibody titers and close contacts [28], [29].

## Discussion

We hypothesized that influenza seroprevalence studies might substantially underestimate IARs if ISP is ignored or based on data from patients presenting to healthcare providers with clinically overt disease. We substantiated this hypothesis with pdmH1N1 seroprevalence data from Hong Kong. To further examine the validity of this conjecture, we performed crude analyses of published pdmH1N1 seroprevalence data from other countries to examine the robustness of their IAR estimates across different seropositivity thresholds and ISP adjustments (see Text S1). In a study in Germany with HI_1∶40_ threshold and no ISP adjustments, Δ*S*_HI 1∶40_/Δ*S*_ HI 1∶20_ was around 0.9, 0.7, and 0.4 among unvaccinated individuals of age 18–32, 33–52 and >52 [8]. Similarly, in a study in New Zealand with HI_1∶40_ threshold and no ISP adjustments, Δ*S*_HI 1∶40_/Δ*S*_ HI 1∶20_ was around 0.7, 0.9, and 0.6 among individuals of age 1–4, 5–19 and 20–59 [6]. Therefore, IAR have probably been underestimated in these studies, especially among older adults.

To our knowledge, only four pdmH1N1 studies had adjusted for ISP: one from the UK with HI_1∶32_ threshold [12], two from the US with HI_1∶40_ threshold [14], [15], and the remaining one by ourselves previously with MN_1∶40_ threshold [13]. All four assumed that ISP was similar to the proportion of patients with clinical disease presenting to healthcare providers who became seropositive. We have already shown that this assumption was inconsistent with population-level seroprevalence rises in Hong Kong where only 60%–70% and 34% of pdmH1N1 infections among age 3–29 and 30–59 became MN_1∶40_ seropositive (Table 1). In the UK study [12], [30], the HI_1∶8_ IAR estimate was 1.2–1.4 times the HI_1∶32_ estimate for those aged 25–44. Similarly, in the study in Florida [14], the HI_1∶20_ IAR estimate was around 1.5–1.7 and 1.9–2.1 times the HI_1∶40_ estimate for those aged 25–49 and 50–64. In the US multi-state study [15], the HI_1∶20_ IAR estimate was 1.2–1.3 times the HI_1∶40_ estimate for those aged 25–64. These results support our conjecture that serologic responses of clinical cases are not necessarily representative.

The most plausible and straightforward explanation was that mild and asymptomatic cases were less likely to become seropositive compared to clinical cases. Testing this hypothesis would require studying serologic responses of infected cases with different severity which would be feasible only with a large prospective cohort study with intensive monitoring to identify mild and asymptomatic cases. Nonetheless, some data from independent studies support this hypothesis. Hung et al reported that among 881 lab-confirmed pdmH1N1 symptomatic patients in Hong Kong, convalescent MN titer correlated well with initial viral load and was independently associated with severity [23]. Specifically, being afrebile on presentation was associated with poorer MN convalescent response. Among 44 RT-PCR confirmed cases (22 cohort subjects with mild symptoms and 22 hospital patients) in Singapore, 89% and 57% became MN_1∶20_ and MN_1∶40_ seropositive [7]. However, there are also published data which contradict the hypothesis. In a study of 24 patients and their 34 household infectees (all RT-PCR confirmed) in Canada, the MN_1∶20_ and MN_1∶40_ seropositivity rates were both 83% [31]. Nonetheless, we caution that titer measurements from different studies might not be directly comparable because serologic titer from different laboratories might vary due to differences in serologic assay protocols and endpoint analysis methods [32]. In particular, serologic follow-ups of clinical cases and seroprevalence studies are often conducted by separate groups with different laboratories, thus adding uncertainty to the consistency between ISP adjustments and seroprevalence data. Our serologic methods were the same as that in the serologic follow-up studies in Hung et al and Mak et al [23], [33], so the results therein should be readily comparable with ours. The Consortium for the Standardization of Influenza Seroepidemiology (CONSISE) is a recent global initiative aiming to standardize both laboratory and field investigation protocols for influenza seroepidemiology (http://consise.tghn.org). Our study suggested that collective interpretation of seroprevalence data and convalescent serologic data should also be an essential part of this standardization effort (e.g. real-time assessment of bias in ISP adjustments by evaluating the consistency of IAR across multiple thresholds and with mixture models; see below for more detailed discussions). Robust sero-surveillance requires an integrated understanding and standardization of the field, laboratory and analytical components of seroepidemiology.

Our results indicated that preexisting MN titers and age group mixing alone could not explain the age distribution of infections. The age-specific susceptibility estimates (Table 1) suggested that older individuals were protected from pdmH1N1 infections by some forms of immunity not reflected by pre-existing MN titers (e.g. cell-mediated immunity). Cytotoxic T cells established by prior seasonal influenza infections were demonstrated to cross react with pdmH1N1 viruses and it is conceivable that such cross-reactive T cell immunity increases with age [34]. Furthermore, the substantial proportion of infections that remained MN_1∶40_ seronegative might have relatively weak and short-lived immunity against pdmH1N1. Waning of such immunity might have subsequently replenished the pool of susceptibles and permitted a second epidemic of pdmH1N1 to occur in Hong Kong in 2011.

Our study has several limitations. First, we assumed that MN titer rises were entirely attributable to pdmH1N1 infection with immunity (lasting until at least 30 November 2009). In theory, it might be possible that individuals could be exposed to pdmH1N1, became MN_1∶20_ seropositive but MN_1∶40_ seronegative, and remained susceptible and noninfectious (i.e. weak serologic response without infection and immunity). This could be an alternative explanation for the discrepancy between Δ*S*_40_/Δ*S*_20_ in seroprevalence data and the ratio of clinical cases that became MN_1∶40_ and MN_1∶20_ seropositive in Hong Kong (Figure 2). In this case, ISP_20_ and ISP_40_ could remain at 1 and 0.9 (as observed among clinical cases) for all infections and the gap between Δ*S*_40_/ISP_40_ and Δ*S*_20_/ISP_20_ would comprise these exposed but uninfected individuals who became MN_1∶20_ seropositive but MN_1∶40_ seronegative. Second, our serologic data were collected via convenience sampling of blood donors, hospital outpatients and participants in community-based studies and hence did not necessarily provide a representative description of pdmH1N1 seroprevalence in the general population. Third, we did not account for any seasonal effects of influenza transmission. The bulk of pdmH1N1 first wave transmission in Hong Kong occurred between 1 September and 30 November 2009, a period during which circulation of seasonal influenza is typically low [35]. As such, the effect of school closure might be stronger than estimated here if seasonality had substantially reduced the transmissibility of pdmH1N1 during September-November 2009. Fourth, we did not consider the potential effect of oseltamivir use on serologic responses. Although oseltamivir use might attenuate serologic response of pdmH1N1 cases [25], treatment coverage was unlikely to be high enough to have a substantial impact on Δ*S*_40_/Δ*S*_20_. Finally, we did not have local social contact data to parameterize our transmission model and had to resort to uncertainty analysis using the POLYMOD matrices. However, this does not imply that we expect the contact pattern in Hong Kong to be similar to that in the European countries. Instead, we showed that our results were robust against the choice of contact matrix because given any contact matrix, the age-specific susceptibility was adjusted by the Bayesian inference algorithm accordingly to result in similar transmission dynamics and hence goodness-of-fit (Figure S4, S5, S6, S7, S8, S9, S10, S11, Figure S12).

Sero-epidemiologic study is the most practical method for accurately estimating influenza IAR, disease severity and population-level immunity which in turn are used to inform vaccination policies and decisions [5]. Our study emphasizes that IAR estimates in seroprevalence studies are sensitive to not only seropositivity thresholds but also ISP adjustments. Steens et al has made a similar observation when they compared pdmH1N1 IAR estimates obtained from conventional thresholds with that from mixture model [17]. Seropositivity thresholds have been typically chosen based on conventions instead of systematic criteria [4]. ISP adjustments have either been ignored or based on clinical patients whose antibody kinetics might not be representative for all infections in the community. Although we have shown that conventional seropositivity thresholds and ISP adjustments have probably led to underestimation of the incidence of pdmH1N1, such bias associated with conventional practice is not specific to pdmH1N1 or the serial cross-sectional design of sero-epidemiology. The longitudinal (cohort) design relies on the definition of seroconversion and infection-seroconversion probability. A recent study by Cauchemez et al reported that under the conventional criterion of seroconversion, namely 4-fold rise or more in antibody titers, influenza IARs were substantially underestimated when there were a significant proportion of subjects with 2-fold rises not explainable by measurement errors alone [36].

These studies and ours thus indicated the need for reevaluating current methods for analyzing influenza serologic data. For example, our group and Baguelin et al previously considered a method for generating real-time estimates of IAR and disease severity for pandemic influenza from serial cross-sectional seroprevalence and clinical surveillance data [12], [13]. This method requires a priori specifying the seropositivity threshold and ISP. Although basing ISP on antibody kinetics of clinical cases is likely to be the best a priori option in the real-time pandemic setting, the associated bias can and should be assessed by evaluating the consistency of IAR and severity estimates across multiple thresholds and with mixture models. A natural extension of the method is to analyze seroprevalence data at multiple thresholds under a Bayesian framework using ISPs among clinical cases as priors (possibly with the extension of integrating transmission dynamics as done here and in Birrel et al [37]). Within this framework, ISP can be continuously updated by the posteriors to reconcile discrepancies between seroprevalence data and ISP priors (e.g. Figure 2). Although the potential bias in ISP priors may not be completely eliminated in real-time, the resulting IAR and severity estimates will likely remain sufficiently precise for informing situational awareness and pandemic responses. In conclusion, our results indicated the need for reexamining conventional practice in influenza sero-epidemiology to develop standards for analyzing influenza serologic data, especially in the context of pandemics when robustness and comparability of IAR estimates are most needed for informing situational awareness and risk assessment. While these studies were conducted within the context of influenza, these methodological approaches are broadly applicable to other infectious disease outbreaks.

## Materials and Methods

### Ethics statement

All study protocols were approved by the Institutional Review Board of The University of Hong Kong/Hospital Authority Hong Kong West Cluster. All adult subjects provided written informed consent, and a parent or guardian of any child participant provided written consent on their behalf.

### Transmission modeling

Major modeling assumptions are summarized below (see Text S1 for further technical details):

1. **Antibody kinetics and testing.** Each infection in age group *a* became MN_1:*X*_ seropositive with probability *ISP_X_*_,*a*_ if they were MN_1:*X*_ seronegative before infection. Because *ISP*_20,*a*_ and *ISP*_40,*a*_ were not simultaneously identifiable from our data, we assumed that *ISP*_20,*a*_ was independent of age. We assumed four pre-pandemic MN titer levels (<1∶10, 1∶10, 1∶20, and ≥1∶40; Figure 1A) and that the *i*th pre-pandemic titer level reduced susceptibility by 1−*g_i_* compared to the lowest level (i.e. *g*_1_ = 1). The onset-to-seropositivity duration was estimated using antibody kinetics data from clinical cases in Hong Kong [33]. Sensitivity (specificity) of serologic testing, defined as the probability that the serologic result was positive (negative) if the specimen was truly seropositive (seronegative), was assumed to be 100%. Imperfect sensitivity and specificity had little impact on our conclusions (see Text S1).
2. **Age-specific susceptibility.** Age group *a* was *h_a_* times as susceptible compared to the 20–29 age group, i.e. *h*_3_ = 1. These age-specific susceptibility parameters modeled differential susceptibility not explainable by the contact matrix and pre-pandemic MN titers.
3. **School closure.** As a proactive mitigation measure, the Hong Kong government closed all kindergartens and primary schools on 11 June 2009 until summer holidays. We assumed that summer holidays and fall semester started on 10 July and 1 September, respectively. Within-age-group mixing was reduced by *π*_0_ for age 3–12 during proactive school closure, and by *π*_1_ and *π*_2_ for age 3–12 and 13–19 during summer holidays.
4. **Importation of infections.** We seeded the pandemic on 1 June 2009 with *M* infectious cases. In addition, we assumed that Hong Kong was subject to an exogenous force of infection that was *ε*_SZ_ times the estimated daily number of lab-confirmed cases in Shenzhen [38] because (i) an average of ∼350,000 people crossed the border on a daily basis; and (ii) sustained low levels of transmission in Hong Kong during November 2009 was likely fueled by the Shenzhen epidemic which peaked in that month [38] (Figure 1B).
5. **Hospitalization.** We assumed that each infection in age group *a* required hospitalization with probability *IHP_a_* (infection-hospitalization probability).
6. **Infectiousness and antibody response.** We assumed that all infected individuals were equally infectious regardless of their antibody response. In the Text S1, we showed that our results were robust against potential association between infectiousness and antibody response.

### Statistical analysis

We fitted the transmission model to the seroprevalence and hospitalization data by estimating the parameters listed in Table 1 using Markov Chain Monte Carlo methods with non-informative flat priors. Because around 85% and 10% of each age group had pre-pandemic MN titer <1∶10 and 1∶10 (Figure 1A), the *g_i_*'s were not identifiable. As such, we assumed *g_i_* = *g^i^* which had negligible effect because the small proportion of individuals who had pre-pandemic titer >1∶10 had little impact on transmission dynamics. Partial rank correlation coefficients (PRCC) among estimated parameters were calculated to identify any strong (defined here as |PRCC|>0.5) but unexpected confounding effects.

For uncertainty analysis, we performed statistical inference for *g* = 0, 0.5 and 1 and each of the nine POLYMOD matrices, i.e. a total of 27 scenarios. Higher *g* (i.e. preexisting MN titer conferred weaker protection) resulted in slightly higher IARs and lower *R*(0) and *T_g_* (Figure S4, S5, S6, S7, S8, S9, S10, S11, Figure S12). Otherwise, all combinations of *g* and **C** resulted in similar goodness-of-fit and parameter estimates except for age-specific susceptibilities. As such, we describe in the main text the inference results (posterior medians and 95% credible intervals) for *g* = 0.5 and **C** = **P_AVG_** unless parameter estimates were sensitive to *g* and **C** (i.e for age-specific susceptibilities).

## Supporting Information

Figure S1
**Probability density function of** 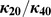 **assuming that** ***sens*** **_20_∼U(0.9,1),** ***spec*** **_40_∼U(0.9,1),** ***sens*** **_40_∼U(0.9,** ***sens*** **_20_),** ***spec*** **_20_∼U(0.9,** ***spec*** **_40_).**
(TIF)

Figure S2 **Estimating the ratio of IAR estimates at higher and lower titers in Baguelin et al.**
(TIF)

Figure S3 **Estimating the ratio of IAR estimates at higher and lower titers in Cox et al and Reed et al. Red, green and blue correspond to assuming the overlap between proportion infected and vaccination coverage was minimal, random and maximal, respectively.**
(TIF)

Figure S4 **Posterior distributions of parameters for different values of g with the average POLYMOD contact matrix.** **A**. Age-dependent parameters including IARs (first column), *ISP*_40_ (second), and age-specific susceptibility (third). **B**. Other parameters including *R*(0), *T_g_*, *ISP*_20_, reduction in within-age-group mixing due to school closure (*π*_0_, *π*_1_, *π*_2_), seed size, and scaling factor for FOI from Shenzhen (*ε_SZ_*). Higher g (i.e. preexisting MN titer conferred weaker protection) resulted in slightly higher IARs and lower *R*(0) and *T_g_*.
(EPS)

Figure S5 **Posterior distributions of parameters for different values of** ***g*** **with the Belgium POLYMOD contact matrix.** **A**. Age-dependent parameters including IARs (first column), *ISP*_40_ (second), and age-specific susceptibility (third). **B**. Other parameters including *R*(0), *T_g_*, *ISP*_20_, reduction in within-age-group mixing due to school closure (*π*_0_, *π*_1_, *π*_2_), seed size, and scaling factor for FOI from Shenzhen (*ε_SZ_*). Higher g (i.e. preexisting MN titer conferred weaker protection) resulted in slightly higher IARs and lower *R*(0) and *T_g_*.
(EPS)

Figure S6 **Posterior distributions of parameters for different values of** ***g*** **with the Finland POLYMOD contact matrix.** **A**. Age-dependent parameters including IARs (first column), *ISP*_40_ (second), and age-specific susceptibility (third). **B**. Other parameters including *R*(0), *T_g_*, *ISP*_20_, reduction in within-age-group mixing due to school closure (*π*_0_, *π*_1_, *π*_2_), seed size, and scaling factor for FOI from Shenzhen (*ε_SZ_*). Higher g (i.e. preexisting MN titer conferred weaker protection) resulted in slightly higher IARs and lower *R*(0) and *T_g_*.
(EPS)

Figure S7 **Posterior distributions of parameters for different values of** ***g*** **with the Germany POLYMOD contact matrix.** **A**. Age-dependent parameters including IARs (first column), *ISP*_40_ (second), and age-specific susceptibility (third). **B**. Other parameters including *R*(0), *T_g_*, *ISP*_20_, reduction in within-age-group mixing due to school closure (*π*_0_, *π*_1_, *π*_2_), seed size, and scaling factor for FOI from Shenzhen (*ε_SZ_*). Higher g (i.e. preexisting MN titer conferred weaker protection) resulted in slightly higher IARs and lower *R*(0) and *T_g_*.
(EPS)

Figure S8 **Posterior distributions of parameters for different values of** ***g*** **with the Italy POLYMOD contact matrix.** **A**. Age-dependent parameters including IARs (first column), *ISP*_40_ (second), and age-specific susceptibility (third). **B**. Other parameters including *R*(0), *T_g_*, *ISP*_20_, reduction in within-age-group mixing due to school closure (*π*_0_, *π*_1_, *π*_2_), seed size, and scaling factor for FOI from Shenzhen (*ε_SZ_*). Higher g (i.e. preexisting MN titer conferred weaker protection) resulted in slightly higher IARs and lower *R*(0) and *T_g_*.
(EPS)

Figure S9 **Posterior distributions of parameters for different values of** ***g*** **with the Luxembourg POLYMOD contact matrix.** **A**. Age-dependent parameters including IARs (first column), *ISP*_40_ (second), and age-specific susceptibility (third). **B**. Other parameters including *R*(0), *T_g_*, *ISP*_20_, reduction in within-age-group mixing due to school closure (*π*_0_, *π*_1_, *π*_2_), seed size, and scaling factor for FOI from Shenzhen (*ε_SZ_*). Higher g (i.e. preexisting MN titer conferred weaker protection) resulted in slightly higher IARs and lower *R*(0) and *T_g_*.
(EPS)

Figure S10 **Posterior distributions of parameters for different values of** ***g*** **with the Netherland POLYMOD contact matrix.** **A**. Age-dependent parameters including IARs (first column), *ISP*_40_ (second), and age-specific susceptibility (third). **B**. Other parameters including *R*(0), *T_g_*, *ISP*_20_, reduction in within-age-group mixing due to school closure (*π*_0_, *π*_1_, *π*_2_), seed size, and scaling factor for FOI from Shenzhen (*ε_SZ_*). Higher g (i.e. preexisting MN titer conferred weaker protection) resulted in slightly higher IARs and lower *R*(0) and *T_g_*.
(EPS)

Figure S11 **Posterior distributions of parameters for different values of** ***g*** **with the Poland POLYMOD contact matrix.** **A**. Age-dependent parameters including IARs (first column), *ISP*_40_ (second), and age-specific susceptibility (third). **B**. Other parameters including *R*(0), *T_g_*, *ISP*_20_, reduction in within-age-group mixing due to school closure (*π*_0_, *π*_1_, *π*_2_), seed size, and scaling factor for FOI from Shenzhen (*ε_SZ_*). Higher g (i.e. preexisting MN titer conferred weaker protection) resulted in slightly higher IARs and lower *R*(0) and *T_g_*.
(EPS)

Figure S12 **Posterior distributions of parameters for different values of** ***g*** **with the United Kingdom POLYMOD contact matrix.** **A**. Age-dependent parameters including IARs (first column), *ISP*_40_ (second), and age-specific susceptibility (third). **B**. Other parameters including *R*(0), *T_g_*, *ISP*_20_, reduction in within-age-group mixing due to school closure (*π*_0_, *π*_1_, *π*_2_), seed size, and scaling factor for FOI from Shenzhen (*ε_SZ_*). Higher g (i.e. preexisting MN titer conferred weaker protection) resulted in slightly higher IARs and lower *R*(0) and *T_g_*.
(EPS)

Table S1 **Model parameters and their posterior statistics comparing base case model and model including the 0–2 and ≥60 age groups.**
(DOCX)

Table S2 **The proportion of infections that were lab-confirmed and hospitalized during the first wave of pdmH1N1 in Hong Kong.**
(DOCX)

Table S3 **Estimating IAR in Baguelin et al using HI 1∶8, 1∶16 and 1∶32 as the seropositivity threshold.**
(DOCX)

Table S4 **Estimating IAR in Cox et al and Reed et al using HI 1∶20 and 1∶40 as the seropositivity threshold.**
(DOCX)

Table S5 **Estimating IAR in Dudareva et al using HI 1∶10, 1∶20 and 1∶40 as the seropositivity threshold.**
(DOCX)

Table S6 **Estimating IAR in Bandaranayake et al using HI 1∶20 and 1∶40 as the seropositivity threshold.**
(DOCX)

Text S1 **Details on the transmission model, statisitcal inference, sensitivity analyses and analysis of consistency of IAR estimates in other seroprevalence studies.**
(DOCX)
