# Supplementary figures and images for: Inferring Influenza Infection Attack Rate from Seroprevalence Data
Source: PLoS Pathog. 2014 Apr 3;10(4):e1004054. doi: 10.1371/journal.ppat.1004054 (PMC3974861; doi:10.1371/journal.ppat.1004054)

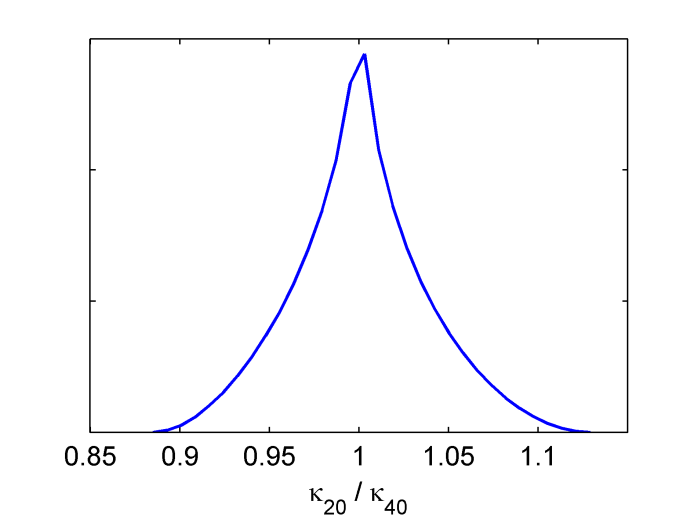

Supplement: Figure S1 — Probability density function of assuming that sens 20∼U(0.9,1), spec 40∼U(0.9,1), sens 40∼U(0.9, sens 20), spec 20∼U(0.9, spec 40). (TIF) [file ppat.1004054.s001.tif]

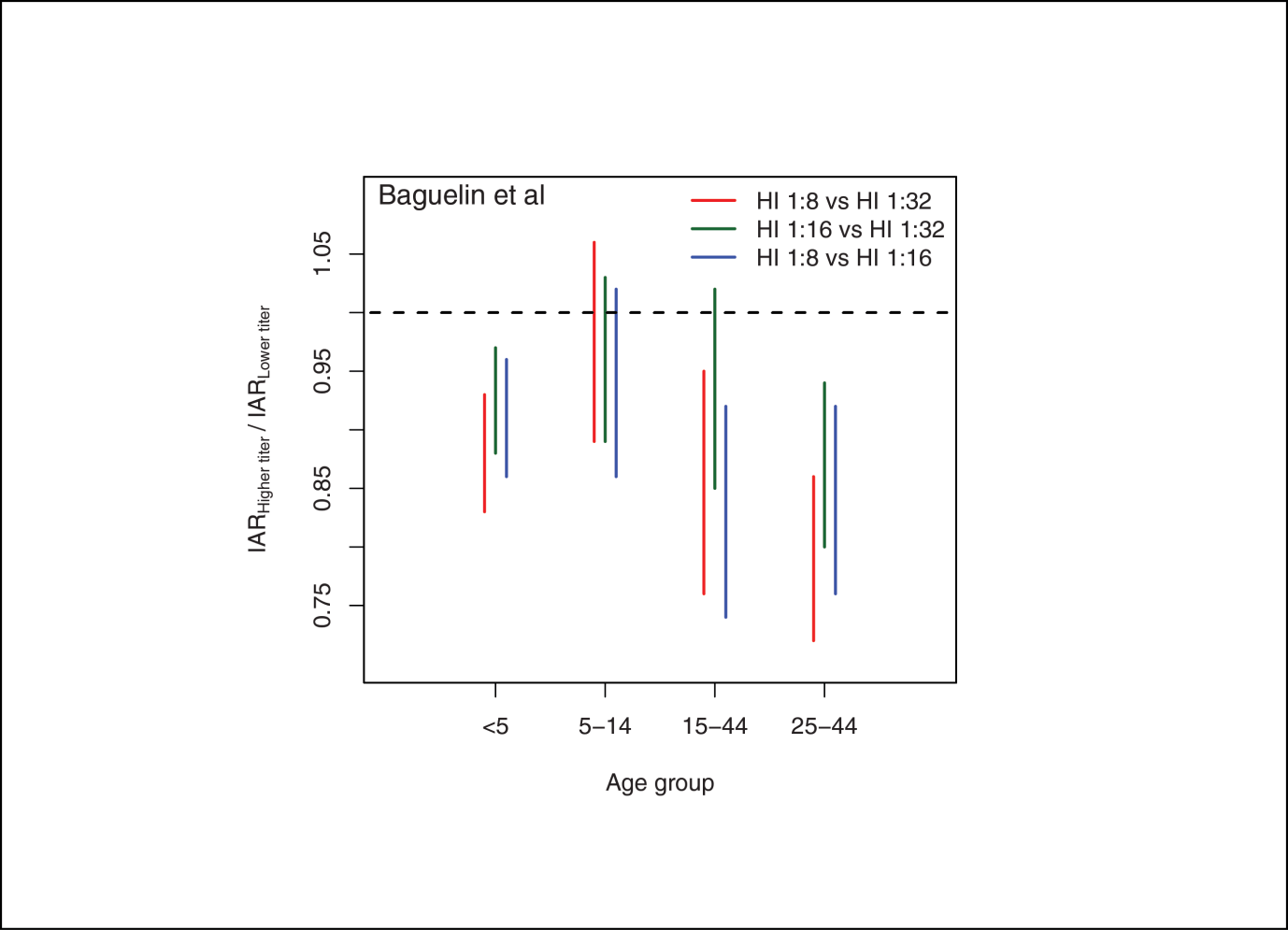

Supplement: Figure S2 — Estimating the ratio of IAR estimates at higher and lower titers in Baguelin et al. (TIF) [file ppat.1004054.s002.tif]

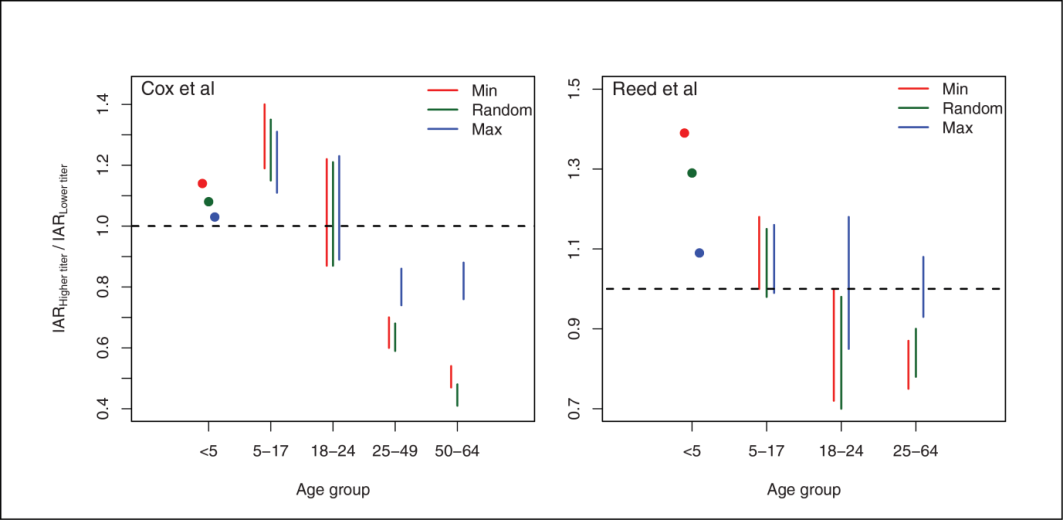

Supplement: Figure S3 — Estimating the ratio of IAR estimates at higher and lower titers in Cox et al and Reed et al. Red, green and blue correspond to assuming the overlap between proportion infected and vaccination coverage was minimal, random and maximal, respectively. (TIF) [file ppat.1004054.s003.tif]
