# Supplementary material for: Inferring Influenza Infection Attack Rate from Seroprevalence Data
Source: PLoS Pathog. 2014 Apr 3;10(4):e1004054. doi: 10.1371/journal.ppat.1004054 (PMC3974861; doi:10.1371/journal.ppat.1004054)
Supplement: Table S1 — Model parameters and their posterior statistics comparing base case model and model including the 0–2 and ≥60 age groups. (DOCX) [file ppat.1004054.s013.docx]

| **Parameter** | **Description** | **Posterior median (95% credible interval)  in the base case model** | **Posterior median (95% credible interval)  with the inclusion of the 0-2 and ≥60 age group** |
| --- | --- | --- | --- |
| *R*(0) | Initial reproductive number | 1.28 (1.23-1.34) | 1.26 (1.23-1.29) |
| *T_g_* | Mean generation time (days) | 2.4 (2.1-2.8) | 2.3 (2.1-2.5) |
| *π*_0_ | Reduction in within-group transmission for the 3-12 age group during proactive school closure | 86% (44%-99%) | 83% (10%-99%) |
| *π*_1_, *π*_2_ | Reduction in within-group transmission during summer holidays | Age 3-12: 59% (46%-73%)  Age 13-19: 23% (15%-30%) | Age 3-12: 59% (46%-78%)  Age 13-19: 22% (16%-30%) |
| *x_a,i_*(0) | Proportion of age group *a* with the *i*th pre-pandemic titer level | Very similar to the distributions in Figure 1A | Very similar to the distributions in Figure 1A |
| *h*_a_ | Age-specific susceptibility of age group *a* compared to the 20-29 age group | Age 3-12: 2.3 (2-2.6)  Age 13-19: 1.3 (1.1-1.5)  Age 30-59: 0.6 (0.5-0.7) | Age 3-12: 2.3 (2-2.6)  Age 13-19: 1.3 (1.1-1.5)  Age 30-59: 0.6 (0.5-0.7) |
| *ISP*_20_ | MN_1:20_ infection-seropositivity probability | 0.99 (0.93-1) | 0.99 (0.95-1) |
| *ISP*_40_*_,a_* | Age-specific MN_1:40_ infection-seropositivity probability | Age 3-12: 0.72 (0.63-0.82)  Age 13-19: 0.65 (0.56-0.75)  Age 20-29: 0.58 (0.49-0.68)  Age 30-59: 0.34 (0.24-0.44) | Age 3-12: 0.68 (0.61-0.76)  Age 13-19: 0.63 (0.55-0.70)  Age 20-29: 0.55 (0.47-0.65)  Age 30-59: 0.32 (0.23-0.41) |
| *µ_Seropos, X_* | Mean delay (days) from onset to MN_1:_*_X_* seropositivity for those infections who became MN_1:_*_X_* seropositive during convalescence | MN_1:20_: 7.3 (6.1-8.6)  MN_1:40_: 9.7 (7.9-11.3) | MN_1:20_: 7.2 (6.1-8.6)  MN_1:40_: 9.7 (7.9-11.3) |
| *M* | Seed size | 246 (132-420) | 243 (91-415) |
| *ε_SZ_* | Scaling factor for exogenous FOI from Shenzhen | 15 (9-23) | 7.6 (6.3-9.1) |
| *IAR_a_* | Infection attack rates | Age 3-12: 52% (46%-58%)  Age 13-19: 49% (43%-56%)  Age 20-29: 25% (21%-29%)  Age 30-59: 13% (10%-16%) | Age 0-2: 30% (27%-33%)  Age 3-12: 55% (50%-59%)  Age 13-19: 52% (48%-56%)  Age 20-29: 26% (22%-29%)  Age 30-59: 14% (12%-17%)  Age ≥60: 7% (6%-9%) |

**Table S1. Model parameters and their posterior statistics comparing base case model and model including the 0-2 and ≥60 age groups.**
