# Supplementary material for: Inferring Influenza Infection Attack Rate from Seroprevalence Data
Source: PLoS Pathog. 2014 Apr 3;10(4):e1004054. doi: 10.1371/journal.ppat.1004054 (PMC3974861; doi:10.1371/journal.ppat.1004054)
Supplement: Table S2 — The proportion of infections that were lab-confirmed and hospitalized during the first wave of pdmH1N1 in Hong Kong. (DOCX) [file ppat.1004054.s014.docx]

| **Age group** | **The proportion of infections lab-confirmed in Hong Kong** | **The proportion of infections hospitalized in Hong Kong** |
| --- | --- | --- |
| 3-12 | 3.5% (3.2%-4%) | 0.89% (0.8%-1%) |
| 13-19 | 2.1% (1.9-2.4%) | 0.29% (0.26%-0.34%) |
| 20-29 | 1.9% (1.6%-2.2%) | 0.22% (0.18%-0.26%) |
| 30-59 | 1.1% (0.9%-1.4%) | 0.23% (0.19%-0.29%) |

**Table S2. The proportion of infections that were lab-confirmed and hospitalized during the first wave of pdmH1N1 in Hong Kong.**
