# Supplementary material for: Inferring Influenza Infection Attack Rate from Seroprevalence Data
Source: PLoS Pathog. 2014 Apr 3;10(4):e1004054. doi: 10.1371/journal.ppat.1004054 (PMC3974861; doi:10.1371/journal.ppat.1004054)
Supplement: Table S3 — Estimating IAR in Baguelin et al using HI 1∶8, 1∶16 and 1∶32 as the seropositivity threshold. (DOCX) [file ppat.1004054.s015.docx]

| Age | Pre second wave in 2009 [[9](#_ENREF_9)] | | | | Post second wave in 2010 [[9](#_ENREF_9)] | | | | Infection- seropositivity probability [[10](#_ENREF_10)] | | | IAR estimates among pre-pandemic seronegatives (%) | | |
| --- | --- | --- | --- | --- | --- | --- | --- | --- | --- | --- | --- | --- | --- | --- |
|  | Total | Seroprevalence (%) | | | Total | Seroprevalence (%) | | | *ISP*_8_ | *ISP*_16_ | *ISP*_32_ | *IAR*_8-_ | *IAR*_16-_ | *IAR*_32-_ |
|  |  | *S*_8,0_ | *S*_16,0_ | *S*_32,0_ |  | *S*_8_ | *S*_16_ | *S*_32_ |  |  |  |  |  |  |
| <5 | 109 | 11 | 9.2 | 9.2 | 196 | 40 | 37 | 35 | 0.93 | 0.93 | 0.89 | 35 | 33 | 32 |
| 5-14 | 171 | 16 | 14 | 14 | 324 | 67 | 66 | 65 | 0.93 | 0.93 | 0.89 | 65 | 65 | 67 |
| 15-24 | 163 | 20 | 17 | 15 | 240 | 54 | 49 | 46 | 0.93 | 0.93 | 0.89 | 46 | 41 | 41 |
| 25-44 | 244 | 17 | 15 | 12 | 470 | 46 | 42 | 36 | 0.93 | 0.93 | 0.89 | 38 | 34 | 31 |

**Table S3. Estimating IAR in Baguelin et al using HI 1:8, 1:16 and 1:32 as the seropositivity threshold.**
