# Supplementary material for: Inferring Influenza Infection Attack Rate from Seroprevalence Data
Source: PLoS Pathog. 2014 Apr 3;10(4):e1004054. doi: 10.1371/journal.ppat.1004054 (PMC3974861; doi:10.1371/journal.ppat.1004054)
Supplement: Table S4 — Estimating IAR in Cox et al and Reed et al using HI 1∶20 and 1∶40 as the seropositivity threshold. (DOCX) [file ppat.1004054.s016.docx]

| Age | Pre-wave [[11](#_ENREF_11)] | | | Post-wave [[7](#_ENREF_7),[11](#_ENREF_11)] | | | | | | Vaccine coverage *P_vax_* (%) | | Vaccination seropositivity probability [[12](#_ENREF_12)] | | Infection seropositivity probability [[6](#_ENREF_6)] | |
| --- | --- | --- | --- | --- | --- | --- | --- | --- | --- | --- | --- | --- | --- | --- | --- |
|  | Total | Seroprevalence (%) | | Cox et al [[7](#_ENREF_7)] | | | Reed et al [[11](#_ENREF_11)] | | | Cox et al [[7](#_ENREF_7)] | Reed et al [[11](#_ENREF_11)] | *VSP*_20_ | *VSP*_40_ | *ISP*_20_ | *ISP*_40_ |
|  |  | *S*_20,0_ | *S*_40,0_ | Total | Seroprevalence (%) | | Total | Seroprevalence (%) | |  |  |  |  |  |  |
|  |  |  |  |  | *S*_20_ | *S*_40_ |  | *S*_20_ | *S*_40_ |  |  |  |  |  |  |
| <5 | 45 | 0 | 0 | 60 | 33 | 28 | 325 | 41 | 37 | 17 | 30 | 0.8 | 0.6 | 0.92 | 0.75 |
| 5-17 | 273 | 15 | 10 | 159 | 49 | 46 | 500 | 70 | 62 | 15 | 25 | 0.95 | 0.8 | 0.92 | 0.75 |
| 18-24 | 95 | 28 | 19 | 150 | 49 | 40 | 454 | 55 | 45 | 6.0 | 21 | 1 | 0.95 | 0.92 | 0.75 |
| 25-49 | 511 | 14 | 6.8 | 169 | 33 | 20 | 963 | 35 | 26 | 6.0 | 15 | 1 | 0.95 | 0.92 | 0.75 |
| 50-64 |  |  |  | 173 | 27 | 16 |  |  |  | 7.4 |  | 1 | 0.95 | 0.92 | 0.75 |
| >64 | 217 | 34 | 16 | 165 | 34 | 18 | 513 | 43 | 28 | 9.8 | 11 | 0.95 | 0.85 | 0.92 | 0.75 |

**Table S4. Estimating IAR in Cox et al and Reed et al using HI 1:20 and 1:40 as the seropositivity threshold.**
