# Supplementary material for: Inferring Influenza Infection Attack Rate from Seroprevalence Data
Source: PLoS Pathog. 2014 Apr 3;10(4):e1004054. doi: 10.1371/journal.ppat.1004054 (PMC3974861; doi:10.1371/journal.ppat.1004054)
Supplement: Table S5 — Estimating IAR in Dudareva et al using HI 1∶10, 1∶20 and 1∶40 as the seropositivity threshold. (DOCX) [file ppat.1004054.s017.docx]

| Age | Pre-pandemic (Nov 2008 to Apr 2009) | | | | Post-pandemic  (Jan 2010 to Apr 2010) | | | | Seroprevalence rise (%) | | | Ratio of seroprevalence rise of higher titer to that of lower titer | | |
| --- | --- | --- | --- | --- | --- | --- | --- | --- | --- | --- | --- | --- | --- | --- |
|  | Total | Seroprevalence (%) | | | Total | Seroprevalence (%) | | | Δ*S*_10_ | Δ*S*_20_ | Δ*S*_40_ | Δ*S*_40_/ Δ*S*_10_ | Δ*S*_40_/ Δ*S*_20_ | Δ*S*_20_/ Δ*S*_10_ |
|  |  | *S*_10,0_ | *S*_20,0_ | *S*_40,0_ |  | *S*_10_ | *S*_20_ | *S*_40_ |  |  |  |  |  |  |
| 18-32 | 144 | 21 | 15 | 12 | 80 | 54 | 48 | 41 | 33 | 33 | 29 | 0.89 | 0.89 | 1.00 |
| 33-52 | 264 | 10 | 6 | 3 | 125 | 33 | 27 | 18 | 23 | 22 | 15 | 0.65 | 0.68 | 0.95 |
| >52 | 437 | 12 | 6 | 3 | 129 | 22 | 13 | 6 | 10 | 7 | 3 | 0.31 | 0.41 | 0.74 |

**Table S5: Estimating IAR in Dudareva et al using HI 1:10, 1:20 and 1:40 as the seropositivity threshold.**
