# Supplementary material for: Inferring Influenza Infection Attack Rate from Seroprevalence Data
Source: PLoS Pathog. 2014 Apr 3;10(4):e1004054. doi: 10.1371/journal.ppat.1004054 (PMC3974861; doi:10.1371/journal.ppat.1004054)
Supplement: Table S6 — Estimating IAR in Bandaranayake et al using HI 1∶20 and 1∶40 as the seropositivity threshold. (DOCX) [file ppat.1004054.s018.docx]

|  | Pre-pandemic  (2004 – Apr 2009) | | | Post-pandemic  (Nov 2009 – Mar 2010) | | | Seroprevalence rise (%) | | Ratio |
| --- | --- | --- | --- | --- | --- | --- | --- | --- | --- |
| Age | Total | Seroprevalence (%) | | Total | Seroprevalence (%) | | Δ*S*_20_ | Δ*S*_40_ | Δ*S*_40_/ Δ*S*_20_ |
|  |  | *S*_20,0_ | *S*_40,0_ |  | *S*_20_ | *S*_40_ |  |  |  |
| 1-4 | 84 | 13 | 6 | 148 | 58 | 37 | 45 | 31 | 0.69 |
| 5-19 | 100 | 26 | 14 | 206 | 65 | 50 | 39 | 35 | 0.90 |
| 20-59 | 213 | 16 | 7 | 479 | 45 | 24 | 29 | 17 | 0.57 |
| ≥60 | 124 | 43 | 23 | 314 | 49 | 23 | 26 | 0 | 0 |

**Table S6: Estimating IAR in Bandaranayake et al using HI 1:20 and 1:40 as the seropositivity threshold.**
