# Supplementary material for: Inferring Influenza Infection Attack Rate from Seroprevalence Data
Source: PLoS Pathog. 2014 Apr 3;10(4):e1004054. doi: 10.1371/journal.ppat.1004054 (PMC3974861; doi:10.1371/journal.ppat.1004054)
Supplement: Text S1 — Details on the transmission model, statisitcal inference, sensitivity analyses and analysis of consistency of IAR estimates in other seroprevalence studies. (DOCX) [file ppat.1004054.s019.docx]

**Inferring influenza infection attack rate from seroprevalence data**

**Supporting Information**

Joseph T. Wu^1^, Kathy Leung^1^, Ranawaka A.P.M Perera^2^, Daniel K. W. Chu^2^, Cheuk Kwong Lee^3^, Ivan F.N. Hung^4^, Che Kit Lin^3^, Su-Vui Lo^5,6^, Yu-Lung Lau^7^, Gabriel M. Leung^1^, Benjamin J. Cowling^1^, J. S. Malik Peiris^2,8^

1. Department of Community Medicine and School of Public Health, Li Ka Shing Faculty of Medicine, The University of Hong Kong, Hong Kong Special Administrative Region, People’s Republic of China.
2. Centre of Influenza Research and School of Public Health, Li Ka Shing Faculty of Medicine, The University of Hong Kong, Hong Kong Special Administrative Region, People’s Republic of China.
3. Hong Kong Red Cross Blood Transfusion Service, Hospital Authority, Hong Kong Special Administrative Region, People’s Republic of China.
4. Department of Medicine, Li Ka Shing Faculty of Medicine, The University of Hong Kong, Hong Kong Special Administrative Region, People’s Republic of China.
5. Hospital Authority, Hong Kong Special Administrative Region, People’s Republic of China.
6. Food and Health Bureau, Government of the Hong Kong Special Administrative Region, People’s Republic of China.
7. Department of Paediatrics and Adolescent Medicine, Li Ka Shing Faculty of Medicine, The University of Hong Kong, Hong Kong Special Administrative Region, People’s Republic of China.
8. HKU-Pasteur Research Pole, Centre of Influenza Research and School of Public Health, Li Ka Shing Faculty of Medicine, The University of Hong Kong, Hong Kong Special Administrative Region, People’s Republic of China.

Corresponding author and author for reprint requests:

Joseph T. Wu, School of Public Health, Li Ka Shing Faculty of Medicine, The University of Hong Kong, Units 624-7, Cyberport 3, Pokfulam, Hong Kong.

Tel: +852 3906 2009; Fax: +852 3520 1945; email: joewu@hku.hk

Table of Contents

[The Epidemic Model 3](#_Toc375216798)

[Statistical inference 5](#_Toc375216799)

[Partial rank correlation coefficients (PRCC) results 9](#_Toc375216800)

[Robustness of results against sensitivity and specificity of serologic assay 10](#_Toc375216801)

[Robustness of results against the exclusion of the 0-2 and ≥60 age groups in disease transmission 11](#_Toc375216802)

[Scaling factor for FOI from Shenzhen 14](#_Toc375216803)

[Analysis of the consistency of IAR estimates in other seroprevalence studies 15](#_Toc375216804)

[References 32](#_Toc375216805)

# The Epidemic Model

Transmission dynamics were simulated using an SEIR structure

$$\begin{matrix} \frac{dX_{a,i}(t)}{dt}=-X_{a,i}(t)g_{i}h_{a}\beta\sum_{b} C_{ab}(t)\left(I_{b}(t)+\varepsilon_{SZ}A_{SZ}(t)v_{b}\right), \\ \frac{dE_{a}(t)}{dt}=-\sum_{i} \frac{dX_{a,i}(t)}{dt}-\frac{E_{a}(t)}{D_{E}}, \\ \frac{dI_{a}(t)}{dt}=\frac{E_{a}(t)}{D_{E}}-\frac{I_{a}(t)}{D_{I}}, \\ \frac{dR_{a}(t)}{dt}=\frac{I_{a}(t)}{D_{I}}. \end{matrix}$$

with the following specifications:

- *X*, *E*, *I* and *R* were the number of susceptible, latent, infectious, and removed individuals in the population with subscripts *a* and *i* indicating age groups and pre-pandemic titer levels, respectively;
- *A_SZ_*(*t*) was the estimated number of new pdmH1N1 cases observed in Shenzhen pandemic surveillance on day *t*.
- {*C_ab_*(*t*)} was the contact matrix at time *t*;
- *g_i_* was the relative susceptibility associated with the *i*th pre-pandemic titer level compared to the lowest level (MN titer <1:10; *g*_1_ = 1);
- *h_a_* was the relative susceptibility of age group *a* compared to the 20-29 age group (*h*_3_ = 1);
- {*v­_b_*, *b* = 1, ..., 4} was the age distribution of the seeds and importations from Shenzhen which was equal to the right eigenvector associated with the largest eigenvalue of the next generation matrix (which in turn corresponded to the reproductive number). That is, {*v­_b_*, *b* = 1, ..., 4} was the age distribution of new cases associated with the next generation matrix [[1](#_ENREF_1)].
- *D_E_* was the latent duration with a base case value of 0.5 days. To relax the exponential waiting time distribution assumption imposed on disease stage durations by the above differential equation formulation, we divided the latent duration into 2 sub-stages so that the latent duration followed an Erlang-2 distribution instead [[2](#_ENREF_2)]. Results remained almost identical when we varied *D_E_* from 0.5 to 2 days and the number of sub-stages from 2 to 6.
- *D_I_* was the mean infectious duration. Similar to the latent duration, the infectious duration was divided into 3 sub-stages. *D_I_* was derived from the mean generation time *T_g_* using the equation 2(*T_g_*−*D_E_*)*D_I_*/(1+*D_I_*) [[3](#_ENREF_3)].

Let *Y_a,i_*(*τ*) be the number of new infections in age group *a* with pre-pandemic titer level *i* on day *τ*, i.e. *Y_a,i_*(*τ*) = *X_a,i_*(*τ*) − *X_a,i_*(*τ*+1). We assumed that the incubation period was an Erlang-2 distribution with mean 1 day. The number of new onsets in age group *a* with pre-pandemic titer level *i* on day *τ* was$Z_{a,i}(\tau)=\sum_{u=0}^{\tau} Y_{a,i}(\tau-u)f_{Incbuation}(u)$ where *f_Incubation_* was the discretized Erlang-2 pdf. Results remained almost identical when we varied the mean of *f_Incubation_* from 0.5 to 2 days and the number of sub-stages from 2 to 6. The hospitalization *H_a,i_*(*τ*) and seroprevalence *S_X,a_*(*τ*) on day *τ* were derived from *Z_a,i_*(0), ..., *Z_a,i_*(*τ*) as follows:

where *f_Hosp,a_* was the age-specific probability density function (pdf) for the delay between onset and hospitalization as in E-Flu (see Figure 1 in [[4](#_ENREF_4)] for details) and *F_Seropos,X_* was the cumulative distribution function (cdf) for the delay between onset and MN_1_:*_X_* seropositivity for those infected cases that became MN_1_:*_X_* seropositive.

# Statistical inference

The likelihood function was the product of the following components:

*Age-stratified pre-pandemic MN titer data*

Multinomial pdfs:

$$\prod_{a} \left(w_{a}!\prod_{i} \frac{x_{a,i}(0)^{z_{a,i}}}{z_{a,i}!}\right)$$

where

- *w_a_* was the number of serum samples for age group *a*
- *z_a,i_* was the observed number of samples with the *i*th MN titer level
- *x_a,i_*(0) was the proportion of age group *a* whose pre-pandemic MN titer was at the *i*th level in the model.

*Age-stratified hospitalization data*

Poisson pdfs:

$$\prod_{a,\tau} \frac{H_{a}(\tau)^{h_{a,\tau}}e^{-H_{a}(\tau)}}{h_{a,\tau}!}$$

where

- *h_a,τ_* was the observed number of hospitalizations in age group *a* on day *τ*
- *H_a_*(*τ*) was the number of hospitalizations in age group *a* on day *τ* in the model.

*Serial cross-sectional age-stratified seroprevalence*

Multinomial pdfs:

$$\prod_{a,j} n_{a,j}!\frac{\left(1-S_{20,a}(\tau_{j})\right)^{n_{a,j}-s_{20,a,j}}}{(n_{a,j}-s_{20,a,j})!}\frac{\left(S_{20,a}(\tau_{j})-S_{40,a}(\tau_{j})\right)^{s_{20,a,j}-s_{40,a,j}}}{(s_{20,a,j}-s_{40,a,j})!}\frac{\left(S_{40,a}(\tau_{j})\right)^{s_{40,a,j}}}{s_{40,a,j}!}$$

where

- *τ_j_* was the collection time of the *j*th cross-section
- *n_a_*,*_j_* was the number of serum samples for age group *a* in the *j*th cross-section
- *s_20,a_*,,*_j_* and *s_40,a_*,,*_j_* were the number of samples MN_1:20_ and MN_1:40_ seropositive in the jth cross-section
- *S*_20_,*_a_*(*τ_j_*) and *S*_40_,*_a_*(*τ_j_*) were MN_1:20_ and MN_1:40_ seroprevalence at time *τ_j_* in the model.

*Antibody kinetics data from serologic follow-up of clinical cases*

We used the data published in [[5](#_ENREF_5)] to estimate the time it took to reach MN_1:20_ and MN_1:40_ seropositivity after symptoms onset. The method was the same as that in our previous work on pdmH1N1 sero-surveillance [[4](#_ENREF_4)] in which we used the data published in Veguilla et al [[6](#_ENREF_6)] instead. Let *B_X_* denote the group of subjects in [[5](#_ENREF_5)] who were MN_1_:*_X_* seronegative before onset and became MN_1_:*_X_* seropositive during convalescence. Let *T*_X_ be the time from onset to MN_1_:*_X_* seroposivity for individuals in *B_X_*. The likelihood function for the antibody kinetics data was:

$$\begin{matrix} \prod_{j=1}^{4} m_{j}!\frac{(1-\rho_{\geq20,j})^{y_{<20,j}}}{y_{<20,j}!}\frac{(\rho_{\geq20,j}-\rho_{\geq40,j})^{m_{j}-y_{<20,j}-y_{\geq40,j}}}{(m_{j}-y_{<20,j}-y_{\geq40,j})!}\frac{\rho_{\geq40,j}^{y_{\geq40,j}}}{y_{\geq40,j}!} \\ \rho_{X,j}=\rho_{X,0}+(1-\rho_{X,0})\theta_{X}F_{Seropos,X}(t_{j}) \end{matrix}$$

where

- *m_j_* was the number of samples on day *t_j_*
- *y*_<20_,*_j_* was the number of samples with MN titer < 1:20 on day *t_j_*
- *y*_≥40_,*_j_* was the number of samples with MN titer ≥ 1:40 on day *t_j_*
- *ρ_X_*,*_j_* was the probability that a sample on day *t_j_* had MN titer ≥ 1:*X*
- *θ_X_* was the proportion of subjects in *B_X_*
- *F­_Seropos_*,*_X_* was the cdf for *T_X_* with mean *µ_Seropos,X_* and standard deviation *σ_Seropos,X_*

We assumed that the probability distributions of *T*_20_ and *T*_40_ − *T*_20_ were Erlang. Because the variance of *T*_20_ and *T*_40_ − *T*_20_ were not simultaneously identifiable, we assumed that *T*_40_ − *T*_20_ followed an Erlang-2 distribution. Results were almost identical when we varied this pdf from Erlang-1 to Erlang-10. The following pdmH1N1 antibody kinetics data were extracted from [[5](#_ENREF_5)]:

| Days since symptoms onset when serum was collected | 0-3 | 4-7 | 8-14 | 15-21 |
| --- | --- | --- | --- | --- |
| No. of sera tested, *m_j_* | 104 | 60 | 62 | 56 |
| No. of sera with MN titer <1:20, *y*_<20_,*_j_* | 98 | 42 | 12 | 2 |
| No. of sera with MN titer ≥1:40, *y*_≥40_,*_j_* | 1 | 5 | 37 | 47 |
| Assumed collection time (days since symptoms onset), *t_j_* | 1.5 | 5.5 | 11 | 18 |

Using the epidemic model, we estimated the following parameters from the hospitalization, seroprevalence and serologic kinetics data:

| **Parameter** | **Description** |
| --- | --- |
| *R*(0) | Initial reproductive number |
| *T_g_* | Mean generation time (days) |
| *π*_0_ | Reduction in within-group transmission for the 3-12 age group during proactive school closure |
| *π*_1_, *π*_2_ | Reduction in within-group transmission for age 3-12 and age 13-19 during summer holidays |
| *x_a,i_*(0) | Proportion of age group *a* with the *i*th pre-pandemic titer level |
| *h*_a_ | Age-specific susceptibility of age group *a* compared to the 20-29 age group |
| *ISP*_20_ | MN_1:20_ infection-seropositivity probability |
| *ISP*_40_*_,a_* | Age-specific MN_1:40_ infection-seropositivity probability |
| *µ_Seropos,20_ µ_Seropos,40_ σ_Seropos,20_* | Mean and standard deviation of the delay from onset to MN_1_:*_X_* seropositivity for those infections who became MN_1_:*_X_* seropositive during convalescence |
| *θ_X ­­_ρ_X_*_,0_ | Supplementary parameters for estimating serologic kinetics from Mak et al (see description of the likelihood function in the text below) *θ_X_*: Proportion of clinical cases that became MN_1_:*_X_* seropositive during convalescence *ρ_X_*_,0_: Proportion of clinical cases who were MN_1_:*_X_* seropositive before infection |
| *M* | Seed size |
| *ε_SZ_* | Scaling factor for exogenous FOI from Shenzhen |
| *ICP_a_* | Age-specific probability of lab-confirmation given infection |
| *IHP_a_* | Age-specific probability of hospitalization given infection |

We assumed non-informative priors for all parameters and implemented Monte Carlo Markov Chain (MCMC) using the Metropolis algorithm to obtain posterior distributions of the parameters. A random step size was chosen for each parameter at every iteration and the variance of the step size for each parameter was automatically adjusted such that the acceptance proportion was between 30% and 70% for each parameter. The MCMC was run for 100,000 iterations for each parameter and the posterior distributions were compiled from the final 70% of the iterations.

# Partial rank correlation coefficients (PRCC) results

PRCC between all pairs of parameters in Table 1 were calculated from the posterior distributions to identify the following strong confounding among the fitted parameters (defined here as |PRCC| > 0.5):

1. *R*(0) and *T_g_* were positively correlated which is a typical feature of all epidemic models (e.g. in a homogenous mixing model, initial epidemic growth rate = (*R*(0) − 1)/*T_g_*).
2. *T_g_* and *ε_SZ_* were positively correlated because longer *T_g_* could be compensated by higher exogenous FOI to maintain similar incidence.
3. *π*_1_ and *π*_2_ were negatively correlated because these parameters reduced disease transmission within the 3-12 and 13-19 age groups during summer holidays, respectively, and therefore could be partially compensated by each other to maintain similar overall epidemic dynamics.
4. *π*_0_ and seed size were positively correlated because these two parameters together determined pdmH1N1 prevalence during the first month of local transmission and higher *π*_0_ could be compensated by higher seed size to maintain similar prevalence during this period.

# Robustness of results against sensitivity and specificity of serologic assay

Let *Z_X_*(*t*) be the proportion of population that would be classified as MN 1:*X* seropositive by our MN assay. If the sensitivity and specificity of the assay were *sens_X_* and *spec_X_*, then *Z_X_*(*t*) and the true seroprevalence *S_X_*(*t*) were related by $Z_{X}(t)=S_{X}(t)\cdot sens_{X}+\left(1-S_{X}(t)\right)\cdot\left(1-spec_{X}\right)$ which implied $\Delta Z_{X}(t)=\Delta S_{X}(t)\left(sens_{X}+spec_{X}-1\right)$. Hence,

$\frac{\Delta Z_{40}(t)}{\Delta Z_{20}(t)}=\frac{\Delta S_{40}(t)\kappa_{40}}{\Delta S_{20}(t)\kappa_{20}}\Leftrightarrow\frac{\Delta S_{40}(t)}{\Delta S_{20}(t)}=\frac{\Delta Z_{40}(t)\kappa_{20}}{\Delta Z_{20}(t)\kappa_{40}}$

where $\kappa_{X}=sens_{X}+spec_{X}-1$. Within the plausible range of $0.9<sens_{40}<sens_{20}<1$ and $0.9<spec_{20}<spec_{40}<1$, the ratio $\kappa_{20}/\kappa_{40}$ remained mostly between 0.85 and 1.15 (Figure S1).


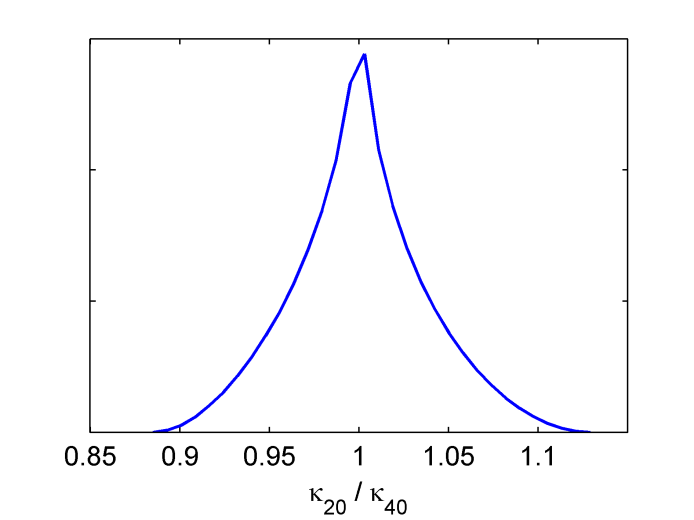


**Figure S1. Probability density function of $\kappa_{20}/\kappa_{40}$assuming that *sens*_20_ ~ U(0.9,1), *spec*_40_ ~ U(0.9,1), *sens*_40_ ~ U(0.9, *sens*_20_), *spec*_20_ ~ U(0.9, *spec*_40_).**

That is, neglecting the imperfectness of sensitivity and specificity would cause our estimates of $\Delta S_{40}/\Delta S_{20}$to deviate from its true value by at most 15%. Therefore, our conclusions, namely that $\Delta S_{40}/\Delta S_{20}$in our serial cross-sectional surveillance data were much lower than *ISP*_40_/*ISP*_20_ = 0.9-1 observed among clinical cases (especially among older age groups), remained valid even if we had taken into account the potential imperfectness of sensitivity and specificity of our MN assay.

# Robustness of results against the exclusion of the 0-2 and ≥60 age groups in disease transmission

In the base case model, the 0-2 yo and ≥60 yo groups were omitted because (i) reliable serologic data from them were not available and (ii) they only represented 2% and 5% of all lab-confirmed pdmH1N1 cases and hospitalizations and thus likely to have small contribution in pdmH1N1 transmission. Omission of these age groups from disease transmission might result in underestimation of IAR. As such, we performed a sensitivity analysis in which we included these two age groups in disease transmission with the following assumptions which tended to result in overestimation of IAR:

1. The 0-2 and 3-12 age groups had the same age-specific susceptibility. If the age-specific susceptibility was significantly higher in the 0-2 age group, then the probability of ICU admission if infected for the 0-2 age group would become lower than that for the 3-12 age group, which seemed implausible. See below for the numerical figures.
2. The 30-59 and ≥60 age groups had the same age-specific susceptibility. The ≥60 age group had higher levels of neutralizing antibody to pdmH1N1 [[5](#_ENREF_5)]. As such, the ≥60 age group was unlikely to be more susceptible to pdmH1N1 infection than the 30-59 age group.

Table S1 shows that the parameter estimates in the base case (i.e. as in Table 1) were very similar to that in this sensitivity analysis. During the study period, there were 3 and 27 pdmH1N1 ICU admissions in the 0-2 and 3-12 age groups, respectively. The sizes of the 0-2 and 3-12 age groups were around 94,000 and 522,300, respectively. The probability of ICU admission if infected that corresponded to the IAR estimates in this sensitivity analysis (Table S1) was 10.5 and 9.4 per 100,000 for the 0-2 and 3-12 age groups, respectively.

| **Parameter** | **Description** | **Posterior median (95% credible interval)  in the base case model** | **Posterior median (95% credible interval)  with the inclusion of the 0-2 and ≥60 age group** |
| --- | --- | --- | --- |
| *R*(0) | Initial reproductive number | 1.28 (1.23-1.34) | 1.26 (1.23-1.29) |
| *T_g_* | Mean generation time (days) | 2.4 (2.1-2.8) | 2.3 (2.1-2.5) |
| *π*_0_ | Reduction in within-group transmission for the 3-12 age group during proactive school closure | 86% (44%-99%) | 83% (10%-99%) |
| *π*_1_, *π*_2_ | Reduction in within-group transmission during summer holidays | Age 3-12: 59% (46%-73%)  Age 13-19: 23% (15%-30%) | Age 3-12: 59% (46%-78%)  Age 13-19: 22% (16%-30%) |
| *x_a,i_*(0) | Proportion of age group *a* with the *i*th pre-pandemic titer level | Very similar to the distributions in Figure 1A | Very similar to the distributions in Figure 1A |
| *h*_a_ | Age-specific susceptibility of age group *a* compared to the 20-29 age group | Age 3-12: 2.3 (2-2.6)  Age 13-19: 1.3 (1.1-1.5)  Age 30-59: 0.6 (0.5-0.7) | Age 3-12: 2.3 (2-2.6)  Age 13-19: 1.3 (1.1-1.5)  Age 30-59: 0.6 (0.5-0.7) |
| *ISP*_20_ | MN_1:20_ infection-seropositivity probability | 0.99 (0.93-1) | 0.99 (0.95-1) |
| *ISP*_40_*_,a_* | Age-specific MN_1:40_ infection-seropositivity probability | Age 3-12: 0.72 (0.63-0.82)  Age 13-19: 0.65 (0.56-0.75)  Age 20-29: 0.58 (0.49-0.68)  Age 30-59: 0.34 (0.24-0.44) | Age 3-12: 0.68 (0.61-0.76)  Age 13-19: 0.63 (0.55-0.70)  Age 20-29: 0.55 (0.47-0.65)  Age 30-59: 0.32 (0.23-0.41) |
| *µ_Seropos, X_* | Mean delay (days) from onset to MN_1_:*_X_* seropositivity for those infections who became MN_1_:*_X_* seropositive during convalescence | MN_1:20_: 7.3 (6.1-8.6)  MN_1:40_: 9.7 (7.9-11.3) | MN_1:20_: 7.2 (6.1-8.6)  MN_1:40_: 9.7 (7.9-11.3) |
| *M* | Seed size | 246 (132-420) | 243 (91-415) |
| *ε_SZ_* | Scaling factor for exogenous FOI from Shenzhen | 15 (9-23) | 7.6 (6.3-9.1) |
| *IAR_a_* | Infection attack rates | Age 3-12: 52% (46%-58%)  Age 13-19: 49% (43%-56%)  Age 20-29: 25% (21%-29%)  Age 30-59: 13% (10%-16%) | Age 0-2: 30% (27%-33%)  Age 3-12: 55% (50%-59%)  Age 13-19: 52% (48%-56%)  Age 20-29: 26% (22%-29%)  Age 30-59: 14% (12%-17%)  Age ≥60: 7% (6%-9%) |

**Table S1. Model parameters and their posterior statistics comparing base case model and model including the 0-2 and ≥60 age groups.**

**Robustness of results against the potential association between infectiousness and antibody response**

In the base case model, we assumed that all exposure leading to a measurable antibody response would result in equally infectious cases. Our results were robust against potential association between infectiousness and antibody response for the following reason. Consider stratifying infected individuals in each age group into two classes: those who would become MN_1:40_ seropositive and those who would not (i.e. MN_1:20_ seropositive but MN_1:40_ seronegative) during convalescence. Let *I_a_*_+_(*t*) and *I_a−_* (*t*) denote their prevalence at time *t* and *h_−_* be the relative infectiousness of those MN_1:20_ seropositive but MN_1:40_ seronegative. Because *I_a_*_+_(*t*) = *ISP*_40_,*_a_I_a_*(*t*) and *I_a−_*(*t*) = (*ISP*_20_ −  *ISP*_40_,*_a_*)*I_a_*(*t*), the force of infection exerted by infected individuals in age group *a* (on any other age group) was proportional to *β*(*I_a_*_+_(*t*) + *h_−_I_a_*_−_(*t*)) = *β*(*ISP*_40_,*_a_* + *h_−_*(*ISP*_20_ −  *ISP*_40_,*_a_*))*I_a_*(*t*) = *β'I_a_*(*t*) where *β'* was a free (composite) parameter and *I_a_*(*t*) was the overall prevalence . The resulting FOI formulation would thus be the same as that in the base case model and result in the same parameter estimates (as in Table 1). Hence, the potential association between infectiousness and antibody had no impact on our results.

# Scaling factor for FOI from Shenzhen

We assumed that the exogenous FOI from Shenzhen was approximately *ε_sz_* times the daily number of lab-confirmed cases in Shenzhen. Intuitively,

$$\varepsilon_{SZ}\approx\frac{\text{proportion of population crossing the border}\times\text{mean infectious duration}}{\text{proportion of pdmH1N1 cases in Shenzhen who were lab-confirmed}}$$

The denominator was used to translate the number of lab-confirmed cases into the total number of infections, i.e. incidence. Mean infectious duration in the numerator was used to translate incidence into prevalence (approximately). Finally, the proportion of population crossing the border in the numerator was used to translate local FOI in Shenzhen into FOI export from Shenzhen into Hong Kong.

Rearranging the above equation gave

$$\begin{matrix} \text{ proportion of pdmH1N1 cases in Shenzhen who were lab-confirmed} \\ \approx\frac{\text{proportion of population crossing the border}\times\text{mean infectious duration}}{\varepsilon_{SZ}} \end{matrix}$$

The population size of Shenzhen is 13 million and an average of around 350,000 people cross the border between Hong Kong and Shenzhen on a daily basis. Using the posterior distributions for Table 1 and the above equation, we estimated that the proportion of pdmH1N1 infections in Shenzhen who were lab-confirmed was 0.39% (0.31%-0.5%). This was plausible in comparison with the proportions of pdmH1N1 cases who were lab-confirmed and hospitalized in Hong Kong in the fitted model (Table S2).

| **Age group** | **The proportion of infections lab-confirmed in Hong Kong** | **The proportion of infections hospitalized in Hong Kong** |
| --- | --- | --- |
| 3-12 | 3.5% (3.2%-4%) | 0.89% (0.8%-1%) |
| 13-19 | 2.1% (1.9-2.4%) | 0.29% (0.26%-0.34%) |
| 20-29 | 1.9% (1.6%-2.2%) | 0.22% (0.18%-0.26%) |
| 30-59 | 1.1% (0.9%-1.4%) | 0.23% (0.19%-0.29%) |

**Table S2. The proportion of infections that were lab-confirmed and hospitalized during the first wave of pdmH1N1 in Hong Kong.**

# Analysis of the consistency of IAR estimates in other seroprevalence studies

We performed a crude analysis of published seroprevalence data from other countries using the following method. For a given seropositivity threshold 1:*X*, let *S_X_*_,0_ and *S_X_* be the pre- and post-study-period seroprevalence. IAR inferred from seroprevalence data under this threshold could be expressed as

$$IAR_{X}=\delta_{X}S_{X,0}IAR_{X-}+\left(1-S_{X,0}\right)IAR_{X-}=IAR_{X-}\left(1-(1-\delta_{X})S_{X,0}\right),$$

where *IAR_X-_* was the IAR among those who were seronegative at the start of the study period and *δ_X_* ∈ [0,1] was the relative susceptibility of those who were seropositive at the start of the study period. We assessed the consistency of IAR estimates from different thresholds using the following bounds on the IAR ratios:

$$\frac{IAR_{\text{Higher titer}-}}{IAR_{\text{Lower titer}-}}\left(1-S_{\text{Higher titer},0}\right)\leq\frac{IAR_{\text{Higher titer}}}{IAR_{\text{Lower titer}}}\leq\frac{IAR_{\text{Higher titer}-}\left(1-S_{\text{Higher titer},0}\right)}{IAR_{\text{Lower titer}-}\left(1-S_{\text{Lower titer},0}\right)}$$

These bounds were obtained using the fact that *δ_X_* and *S_X_*_,0_ both decreased with *X*, hence

$$\begin{matrix} \frac{IAR_{\text{Higher titer}}}{IAR_{\text{Lower titer}}}=\frac{IAR_{\text{Higher titer}-}\left(1-(1-\delta_{\text{Higher titer}})S_{\text{Higher titer},0}\right)}{IAR_{\text{Lower titer}-}\left(1-(1-\delta_{\text{Lower titer}})S_{\text{Lower titer},0}\right)} \\ \geq\frac{IAR_{\text{Higher titer}-}\left(1-S_{\text{Higher titer},0}\right)}{IAR_{\text{Lower titer}-}\left(1-(1-\delta_{\text{Lower titer}})S_{\text{Lower titer},0}\right)}\text{ } \\ \geq\frac{IAR_{\text{Higher titer}-}}{IAR_{\text{Lower titer}-}}\min_{\delta_{\text{Lower titer}}} \frac{1-S_{\text{Higher titer},0}}{\left(1-(1-\delta_{\text{Lower titer}})S_{\text{Lower titer},0}\right)} \\ \geq\frac{IAR_{\text{Higher titer}-}}{IAR_{\text{Lower titer}-}}\left(1-S_{\text{Higher titer},0}\right) \end{matrix}$$

and

$$\begin{matrix} \frac{IAR_{\text{Higher titer}}}{IAR_{\text{Lower titer}}}=\frac{IAR_{\text{Higher titer}-}\left(1-(1-\delta_{\text{Higher titer}})S_{\text{Higher titer},0}\right)}{IAR_{\text{Lower titer}-}\left(1-(1-\delta_{\text{Lower titer}})S_{\text{Lower titer},0}\right)} \\ \leq\frac{IAR_{\text{Higher titer}-}\left(1-(1-\delta_{\text{Lower titer}})S_{\text{Higher titer},0}\right)}{IAR_{\text{Lower titer}-}\left(1-(1-\delta_{\text{Lower titer}})S_{\text{Lower titer},0}\right)}\text{because }\delta_{\text{Higher titer}}<\delta_{\text{Lower titer}} \\ \leq\frac{IAR_{\text{Higher titer}-}}{IAR_{\text{Lower titer}-}}\max_{\delta_{\text{Lower titer}}} \frac{\left(1-(1-\delta_{\text{Lower titer}})S_{\text{Higher titer},0}\right)}{\left(1-(1-\delta_{\text{Lower titer}})S_{\text{Lower titer},0}\right)} \\ \leq\frac{IAR_{\text{Higher titer}-}\left(1-S_{\text{Higher titer},0}\right)}{IAR_{\text{Lower titer}-}\left(1-S_{\text{Lower titer},0}\right)} \end{matrix}$$

IAR estimates from different thresholds were deemed inconsistent if the bounds were tight and did not cover 1. Calculations of these bounds required *IAR_X-_* which was inferred from seroprevalence data as follows:

where *P_vax_* was the vaccination coverage during the study period, *VSP_X_* was the probability of seropositivity if vaccinated (for those who were seronegative before vaccination), and *ϕ* was the overlap between proportion infected and vaccination coverage. We considered three scenarios of such overlap:

In what follows, *P_vax_* = 0 except for the two studies from the US [[7](#_ENREF_7)] .

*Study 1: "Age-Specific Incidence of A/H1N1 2009 Influenza Infection in England from Sequential Antibody Prevalence Data Using Likelihood-Based Estimation" by Baguelin et al*

The serologic data presented in Baguelin et al [[8](#_ENREF_8)] could only be used to infer seroprevalence at HI 1:32 but not at lower titer thresholds. However, the underlying serologic data were the same as that in Hardelid et al [[9](#_ENREF_9)]. As such, we evaluated the consistency of IAR estimates under different thresholds that would have been obtained in Baguelin et al using the seroprevalence data at HI 1:8 and 1:16 contained in Figure 8 of Hardelid et al with *ISP*_32_ = 0.89, *ISP*_16_ = 0.93, *ISP*_8_ = 0.93 [[10](#_ENREF_10)].

| Age | Pre second wave in 2009 [[9](#_ENREF_9)] | | | | Post second wave in 2010 [[9](#_ENREF_9)] | | | | Infection- seropositivity probability [[10](#_ENREF_10)] | | | IAR estimates among pre-pandemic seronegatives (%) | | |
| --- | --- | --- | --- | --- | --- | --- | --- | --- | --- | --- | --- | --- | --- | --- |
|  | Total | Seroprevalence (%) | | | Total | Seroprevalence (%) | | | *ISP*_8_ | *ISP*_16_ | *ISP*_32_ | *IAR*_8-_ | *IAR*_16-_ | *IAR*_32-_ |
|  |  | *S*_8,0_ | *S*_16,0_ | *S*_32,0_ |  | *S*_8_ | *S*_16_ | *S*_32_ |  |  |  |  |  |  |
| <5 | 109 | 11 | 9.2 | 9.2 | 196 | 40 | 37 | 35 | 0.93 | 0.93 | 0.89 | 35 | 33 | 32 |
| 5-14 | 171 | 16 | 14 | 14 | 324 | 67 | 66 | 65 | 0.93 | 0.93 | 0.89 | 65 | 65 | 67 |
| 15-24 | 163 | 20 | 17 | 15 | 240 | 54 | 49 | 46 | 0.93 | 0.93 | 0.89 | 46 | 41 | 41 |
| 25-44 | 244 | 17 | 15 | 12 | 470 | 46 | 42 | 36 | 0.93 | 0.93 | 0.89 | 38 | 34 | 31 |

**Table S3. Estimating IAR in Baguelin et al using HI 1:8, 1:16 and 1:32 as the seropositivity threshold.**


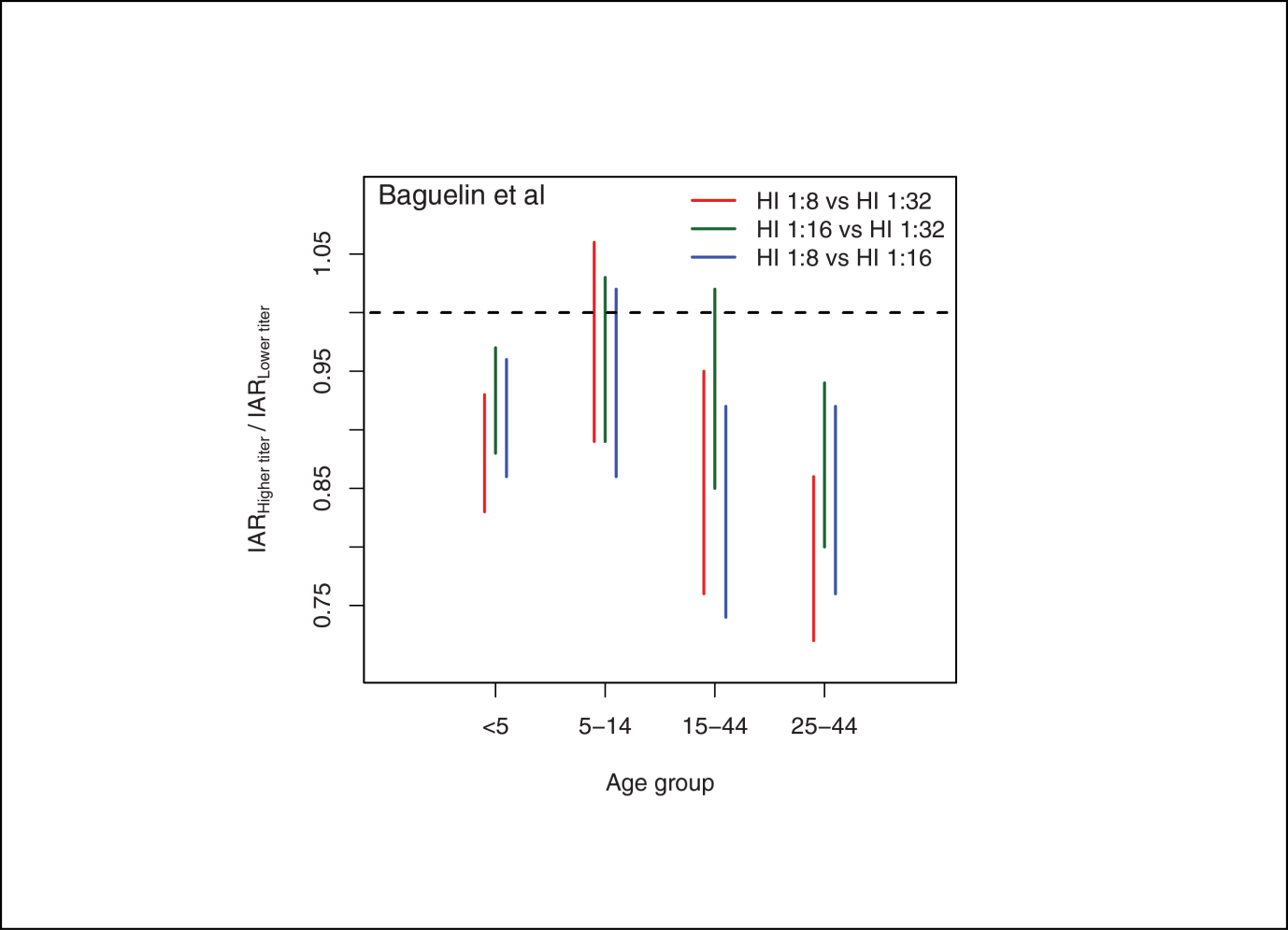


**Figure S2: Estimating the ratio of IAR estimates at higher and lower titers in Baguelin et al.**

*Study 2 and 3:"Prevalence of 2009 pandemic influenza A (H1N1) virus antibodies, Tampa Bay Florida--November-December, 2009" by Cox et al [*[*7*](#_ENREF_7)*] and "Prevalence of Seropositivity to Pandemic Influenza A/H1N1 Virus in the United States following the 2009 Pandemic" by Reed et al [*[*11*](#_ENREF_11)*]*

These studies adopted the definitions of sensitivity and specificity of serologic assays in Veguilla et al [[6](#_ENREF_6)] which were synonymous with ISP and pre-pandemic seroprevalence in our study. Immunogenicity data of pandemic vaccination were taken from Nolan et al [[12](#_ENREF_12)]. The overlap between those who were infected and those who were vaccinated among the 25-49 age group was likely to be far from maximal because both IAR and vaccination coverage were in the range of 10-20% for this age group. As such, when presenting the results in Table S4 and Figure S3 in the discussion section of the main text, we focused on the minimal and random overlap scenarios.

| Age | Pre-wave [[11](#_ENREF_11)] | | | Post-wave [[7](#_ENREF_7),[11](#_ENREF_11)] | | | | | | Vaccine coverage *P_vax_* (%) | | Vaccination seropositivity probability [[12](#_ENREF_12)] | | Infection seropositivity probability [[6](#_ENREF_6)] | |
| --- | --- | --- | --- | --- | --- | --- | --- | --- | --- | --- | --- | --- | --- | --- | --- |
|  | Total | Seroprevalence (%) | | Cox et al [[7](#_ENREF_7)] | | | Reed et al [[11](#_ENREF_11)] | | | Cox et al [[7](#_ENREF_7)] | Reed et al [[11](#_ENREF_11)] | *VSP*_20_ | *VSP*_40_ | *ISP*_20_ | *ISP*_40_ |
|  |  | *S*_20,0_ | *S*_40,0_ | Total | Seroprevalence (%) | | Total | Seroprevalence (%) | |  |  |  |  |  |  |
|  |  |  |  |  | *S*_20_ | *S*_40_ |  | *S*_20_ | *S*_40_ |  |  |  |  |  |  |
| <5 | 45 | 0 | 0 | 60 | 33 | 28 | 325 | 41 | 37 | 17 | 30 | 0.8 | 0.6 | 0.92 | 0.75 |
| 5-17 | 273 | 15 | 10 | 159 | 49 | 46 | 500 | 70 | 62 | 15 | 25 | 0.95 | 0.8 | 0.92 | 0.75 |
| 18-24 | 95 | 28 | 19 | 150 | 49 | 40 | 454 | 55 | 45 | 6.0 | 21 | 1 | 0.95 | 0.92 | 0.75 |
| 25-49 | 511 | 14 | 6.8 | 169 | 33 | 20 | 963 | 35 | 26 | 6.0 | 15 | 1 | 0.95 | 0.92 | 0.75 |
| 50-64 |  |  |  | 173 | 27 | 16 |  |  |  | 7.4 |  | 1 | 0.95 | 0.92 | 0.75 |
| >64 | 217 | 34 | 16 | 165 | 34 | 18 | 513 | 43 | 28 | 9.8 | 11 | 0.95 | 0.85 | 0.92 | 0.75 |

**Table S4. Estimating IAR in Cox et al and Reed et al using HI 1:20 and 1:40 as the seropositivity threshold.**


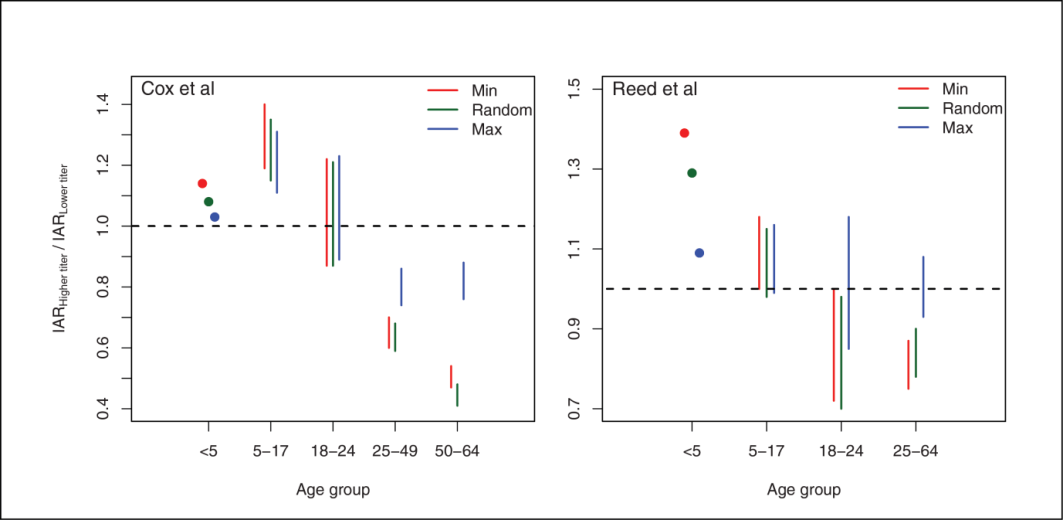


**Figure S3: Estimating the ratio of IAR estimates at higher and lower titers in Cox et al and Reed et al. Red, green and blue correspond to assuming the overlap between proportion infected and vaccination coverage was minimal, random and maximal, respectively.**

*Study 4: " Prevalence of Antibodies to 2009 Pandemic Influenza A (H1N1) Virus in German Adult Population in Pre- and Post-Pandemic Period" by Dudareva et al [*[*13*](#_ENREF_13)*]*

HI_1:40_ seroprevalence data were directly obtained from their Table S4 while HI_1:10_ and HI_1:20_ seroprevalence data were estimated from their Figure 2 and the below Table S5.

| Age | Pre-pandemic (Nov 2008 to Apr 2009) | | | | Post-pandemic  (Jan 2010 to Apr 2010) | | | | Seroprevalence rise (%) | | | Ratio of seroprevalence rise of higher titer to that of lower titer | | |
| --- | --- | --- | --- | --- | --- | --- | --- | --- | --- | --- | --- | --- | --- | --- |
|  | Total | Seroprevalence (%) | | | Total | Seroprevalence (%) | | | Δ*S*_10_ | Δ*S*_20_ | Δ*S*_40_ | Δ*S*_40_/ Δ*S*_10_ | Δ*S*_40_/ Δ*S*_20_ | Δ*S*_20_/ Δ*S*_10_ |
|  |  | *S*_10,0_ | *S*_20,0_ | *S*_40,0_ |  | *S*_10_ | *S*_20_ | *S*_40_ |  |  |  |  |  |  |
| 18-32 | 144 | 21 | 15 | 12 | 80 | 54 | 48 | 41 | 33 | 33 | 29 | 0.89 | 0.89 | 1.00 |
| 33-52 | 264 | 10 | 6 | 3 | 125 | 33 | 27 | 18 | 23 | 22 | 15 | 0.65 | 0.68 | 0.95 |
| >52 | 437 | 12 | 6 | 3 | 129 | 22 | 13 | 6 | 10 | 7 | 3 | 0.31 | 0.41 | 0.74 |

**Table S5: Estimating IAR in Dudareva et al using HI 1:10, 1:20 and 1:40 as the seropositivity threshold.**

*Study 5: "Risk Factors and Immunity in a Nationally Representative Population following the 2009 Influenza A(H1N1) Pandemic" by Bandaranayake et al [*[*14*](#_ENREF_14)*]*

Pre- and post-pandemic HI_1:20_ and HI_1:40_ seroprevalence data were estimated from Table 2 and Figure 2 therein.

|  | Pre-pandemic  (2004 – Apr 2009) | | | Post-pandemic  (Nov 2009 – Mar 2010) | | | Seroprevalence rise (%) | | Ratio |
| --- | --- | --- | --- | --- | --- | --- | --- | --- | --- |
| Age | Total | Seroprevalence (%) | | Total | Seroprevalence (%) | | Δ*S*_20_ | Δ*S*_40_ | Δ*S*_40_/ Δ*S*_20_ |
|  |  | *S*_20,0_ | *S*_40,0_ |  | *S*_20_ | *S*_40_ |  |  |  |
| 1-4 | 84 | 13 | 6 | 148 | 58 | 37 | 45 | 31 | 0.69 |
| 5-19 | 100 | 26 | 14 | 206 | 65 | 50 | 39 | 35 | 0.90 |
| 20-59 | 213 | 16 | 7 | 479 | 45 | 24 | 29 | 17 | 0.57 |
| ≥60 | 124 | 43 | 23 | 314 | 49 | 23 | 26 | 0 | 0 |

**Table S6: Estimating IAR in Bandaranayake et al using HI 1:20 and 1:40 as the seropositivity threshold.**

**
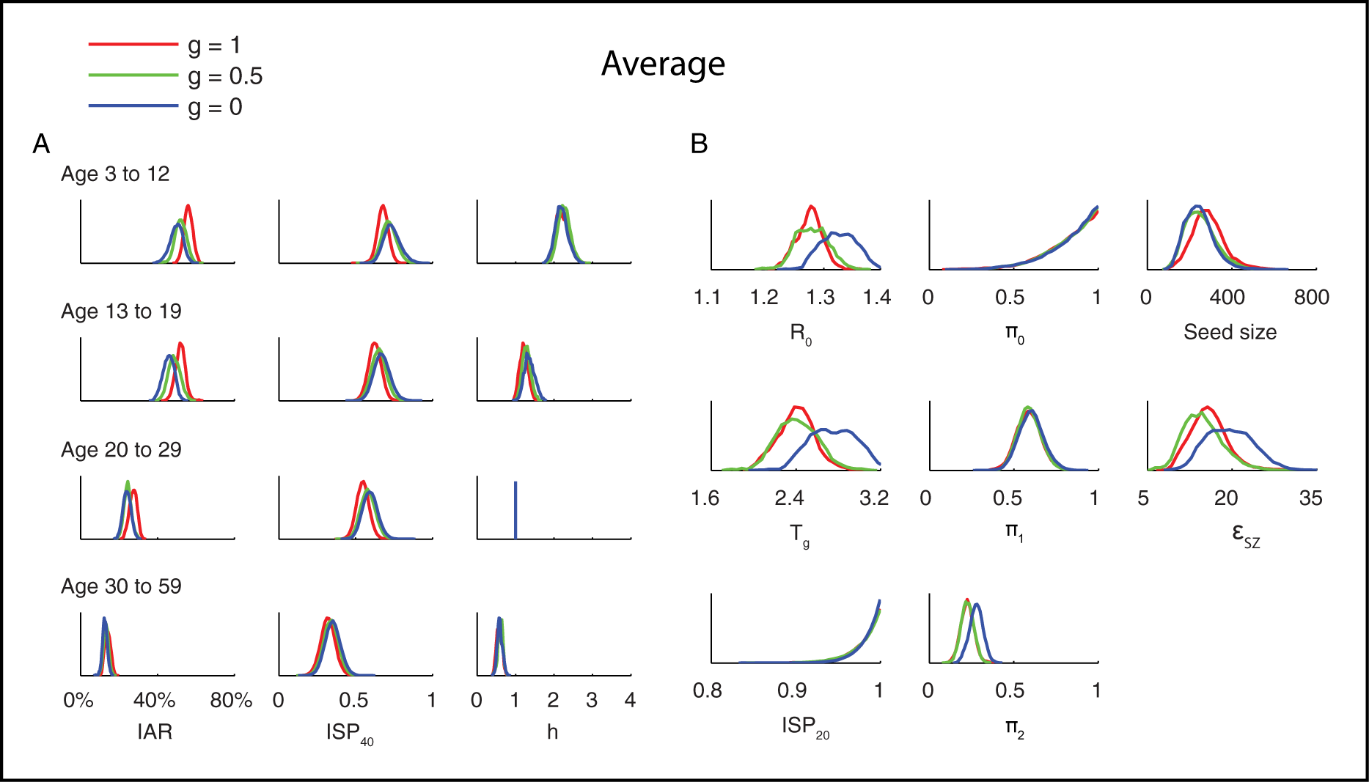
**

**Figure S4. Posterior distributions of parameters for different values of g with the average POLYMOD contact matrix. A.** Age-dependent parameters including IARs (first column), *ISP*_40_ (second), and age-specific susceptibility (third). **B.** Other parameters including *R*(0), *T_g_,* *ISP*_20_, reduction in within-age-group mixing due to school closure (*π*_0_, *π*_1_, *π*_2_), seed size, and scaling factor for FOI from Shenzhen (*ε_SZ_*). **Higher g (i.e. preexisting MN titer conferred weaker protection) resulted in slightly higher IARs and lower *R*(0) and *T_g_*.**

**
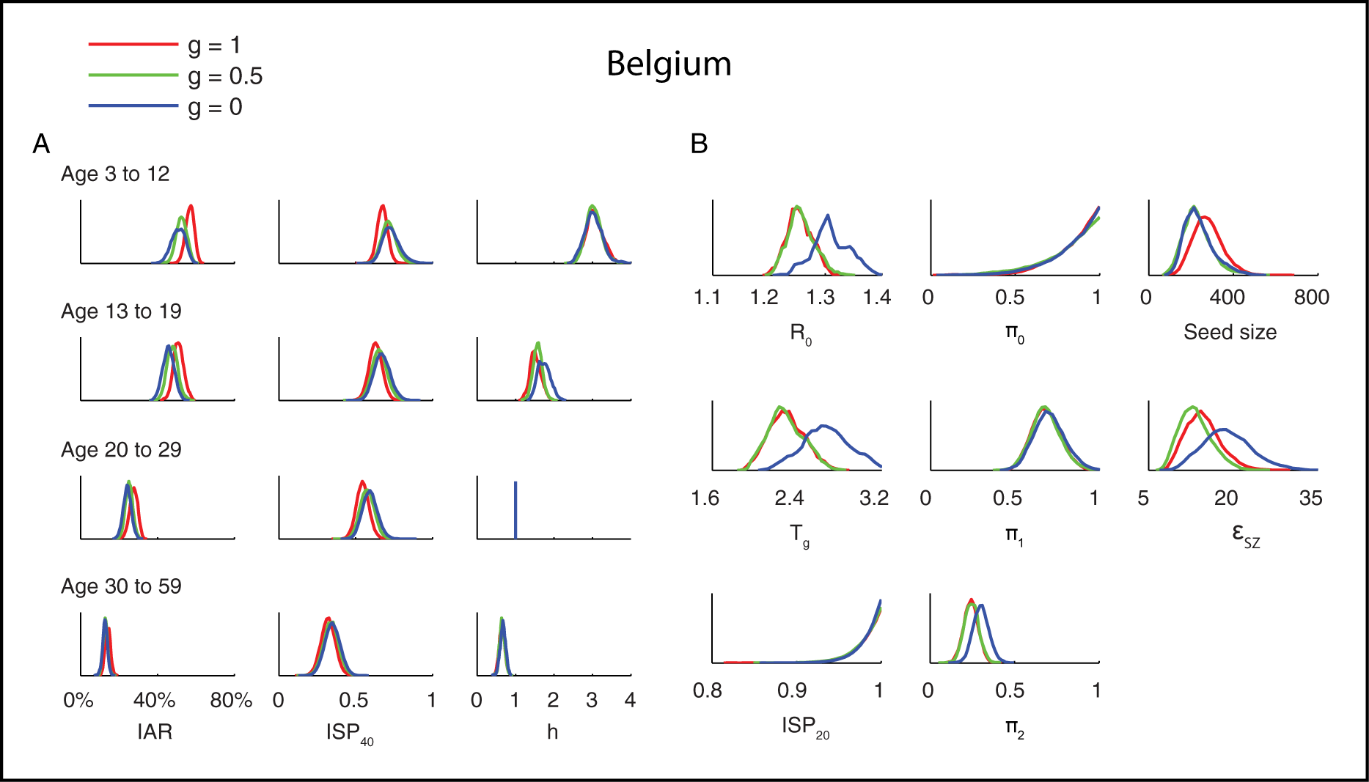
**

**Figure S5. Posterior distributions of parameters for different values of g with the Belgium POLYMOD contact matrix. A.** Age-dependent parameters including IARs (first column), *ISP*_40_ (second), and age-specific susceptibility (third). **B.** Other parameters including *R*(0), *T_g_,* *ISP*_20_, reduction in within-age-group mixing due to school closure (*π*_0_, *π*_1_, *π*_2_), seed size, and scaling factor for FOI from Shenzhen (*ε_SZ_*). **Higher g (i.e. preexisting MN titer conferred weaker protection) resulted in slightly higher IARs and lower *R*(0) and *T_g_*.**

**
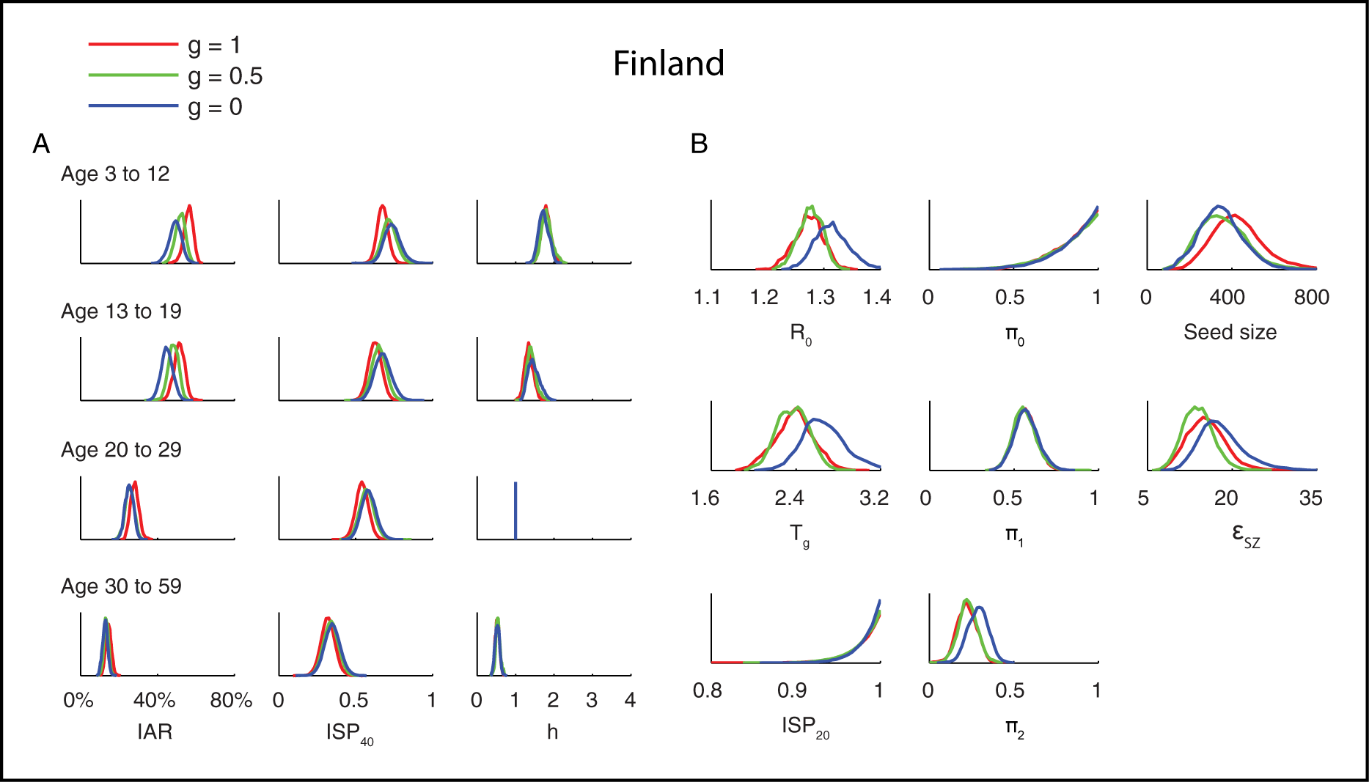
**

**Figure S6. Posterior distributions of parameters for different values of g with the Finland POLYMOD contact matrix. A.** Age-dependent parameters including IARs (first column), *ISP*_40_ (second), and age-specific susceptibility (third). **B.** Other parameters including *R*(0), *T_g_,* *ISP*_20_, reduction in within-age-group mixing due to school closure (*π*_0_, *π*_1_, *π*_2_), seed size, and scaling factor for FOI from Shenzhen (*ε_SZ_*). **Higher g (i.e. preexisting MN titer conferred weaker protection) resulted in slightly higher IARs and lower *R*(0) and *T_g_*.**

**
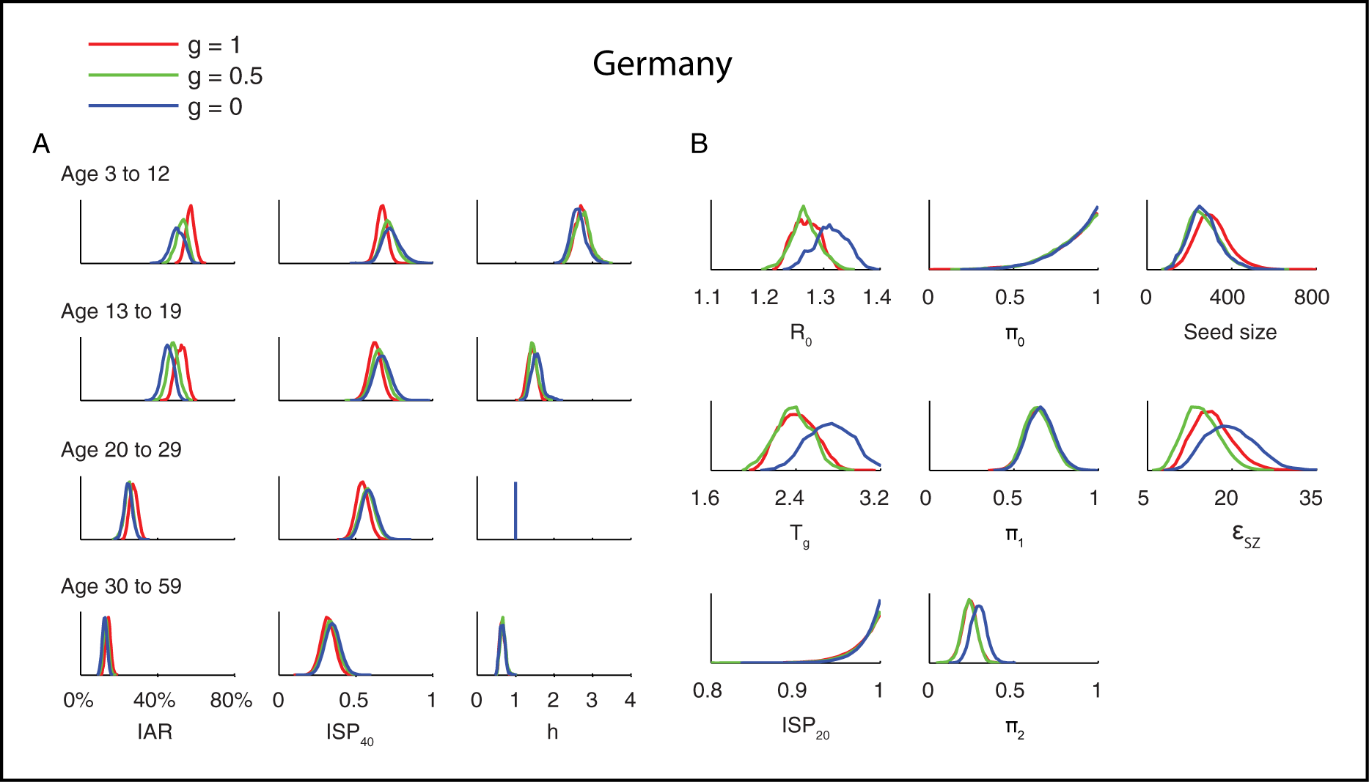
**

**Figure S7. Posterior distributions of parameters for different values of g with the Germany POLYMOD contact matrix. A.** Age-dependent parameters including IARs (first column), *ISP*_40_ (second), and age-specific susceptibility (third). **B.** Other parameters including *R*(0), *T_g_,* *ISP*_20_, reduction in within-age-group mixing due to school closure (*π*_0_, *π*_1_, *π*_2_), seed size, and scaling factor for FOI from Shenzhen (*ε_SZ_*). **Higher g (i.e. preexisting MN titer conferred weaker protection) resulted in slightly higher IARs and lower *R*(0) and *T_g_*.**

**
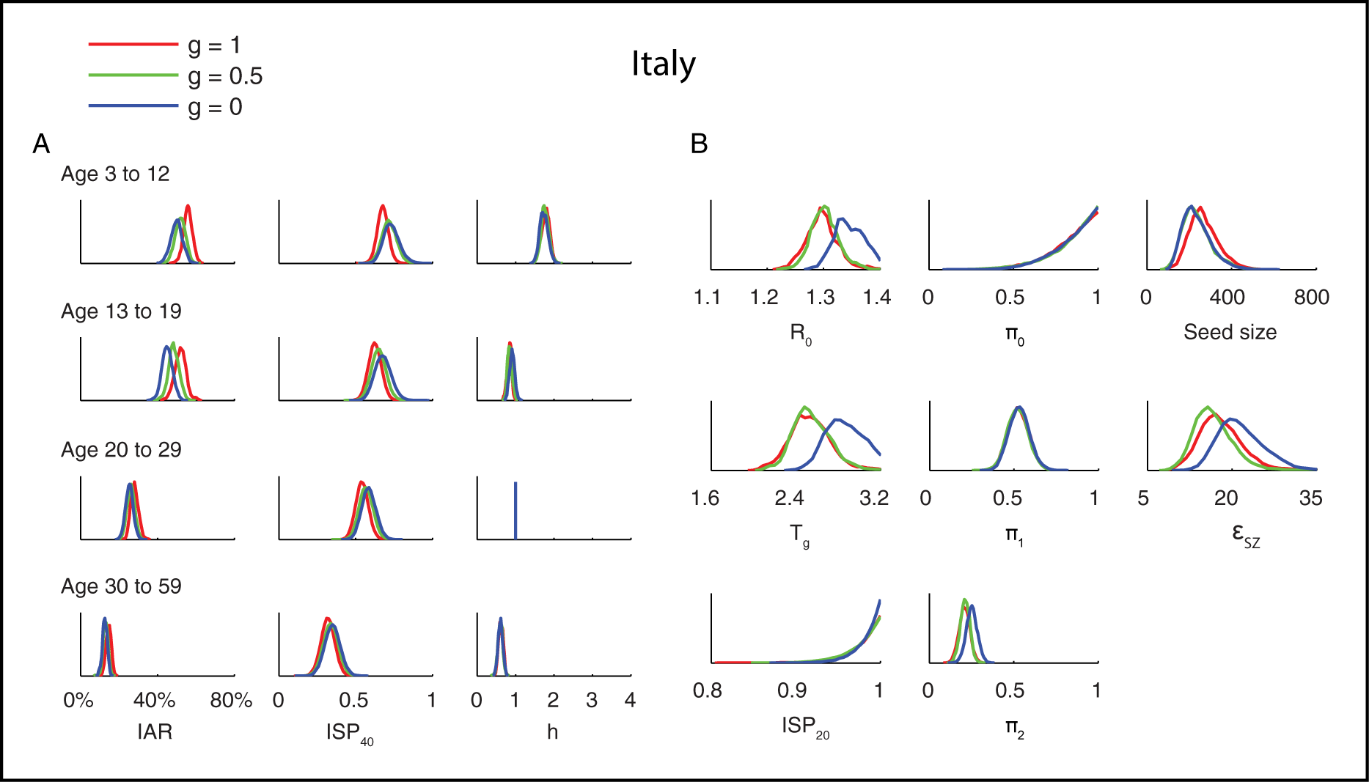
**

**Figure S8. Posterior distributions of parameters for different values of g with the Italy POLYMOD contact matrix. A.** Age-dependent parameters including IARs (first column), *ISP*_40_ (second), and age-specific susceptibility (third). **B.** Other parameters including *R*(0), *T_g_,* *ISP*_20_, reduction in within-age-group mixing due to school closure (*π*_0_, *π*_1_, *π*_2_), seed size, and scaling factor for FOI from Shenzhen (*ε_SZ_*). **Higher g (i.e. preexisting MN titer conferred weaker protection) resulted in slightly higher IARs and lower *R*(0) and *T_g_*.**

**
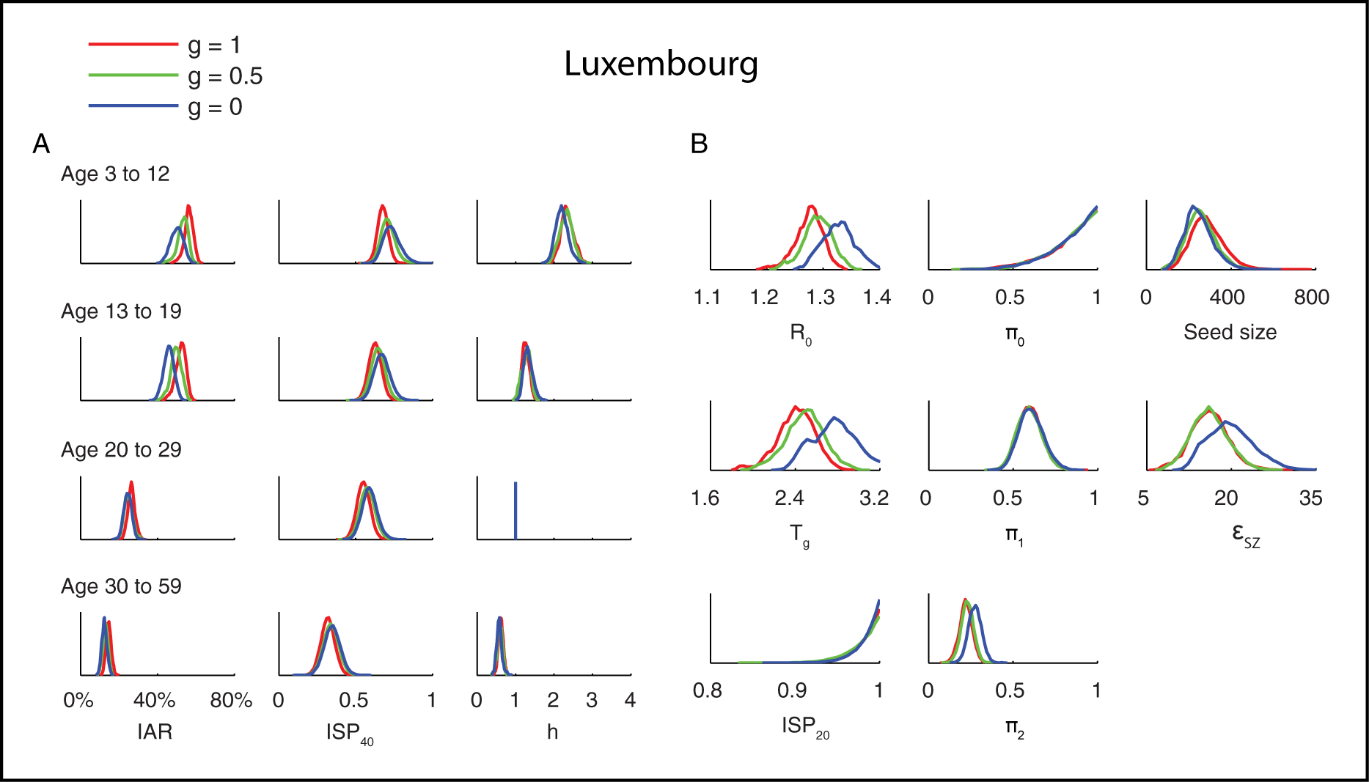
**

**Figure S9. Posterior distributions of parameters for different values of g with the Luxembourg POLYMOD contact matrix. A.** Age-dependent parameters including IARs (first column), *ISP*_40_ (second), and age-specific susceptibility (third). **B.** Other parameters including *R*(0), *T_g_,* *ISP*_20_, reduction in within-age-group mixing due to school closure (*π*_0_, *π*_1_, *π*_2_), seed size, and scaling factor for FOI from Shenzhen (*ε_SZ_*). **Higher g (i.e. preexisting MN titer conferred weaker protection) resulted in slightly higher IARs and lower *R*(0) and *T_g_*.**

**
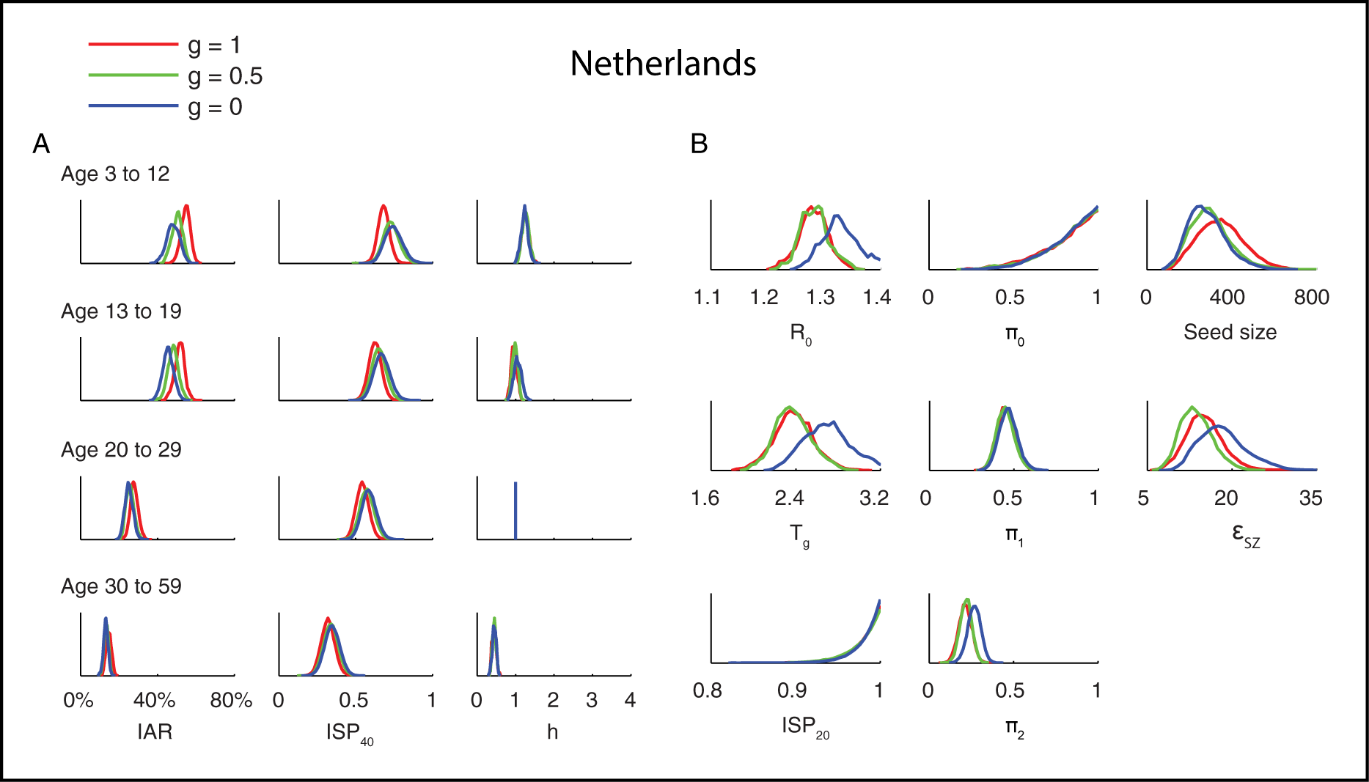
**

**Figure S10. Posterior distributions of parameters for different values of g with the Netherland POLYMOD contact matrix. A.** Age-dependent parameters including IARs (first column), *ISP*_40_ (second), and age-specific susceptibility (third). **B.** Other parameters including *R*(0), *T_g_,* *ISP*_20_, reduction in within-age-group mixing due to school closure (*π*_0_, *π*_1_, *π*_2_), seed size, and scaling factor for FOI from Shenzhen (*ε_SZ_*). **Higher g (i.e. preexisting MN titer conferred weaker protection) resulted in slightly higher IARs and lower *R*(0) and *T_g_*.**

**
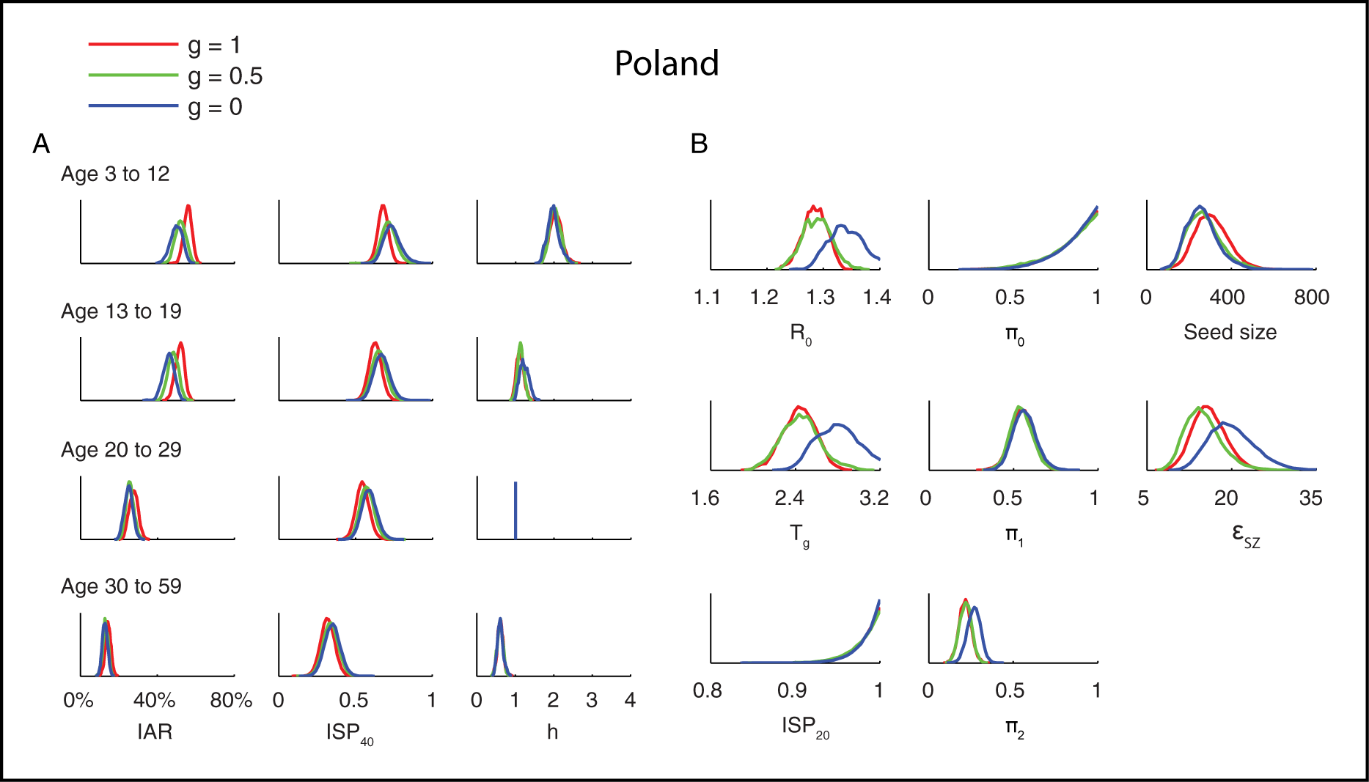
**

**Figure S11. Posterior distributions of parameters for different values of g with the Poland POLYMOD contact matrix. A.** Age-dependent parameters including IARs (first column), *ISP*_40_ (second), and age-specific susceptibility (third). **B.** Other parameters including *R*(0), *T_g_,* *ISP*_20_, reduction in within-age-group mixing due to school closure (*π*_0_, *π*_1_, *π*_2_), seed size, and scaling factor for FOI from Shenzhen (*ε_SZ_*). **Higher g (i.e. preexisting MN titer conferred weaker protection) resulted in slightly higher IARs and lower *R*(0) and *T_g_*.**

**
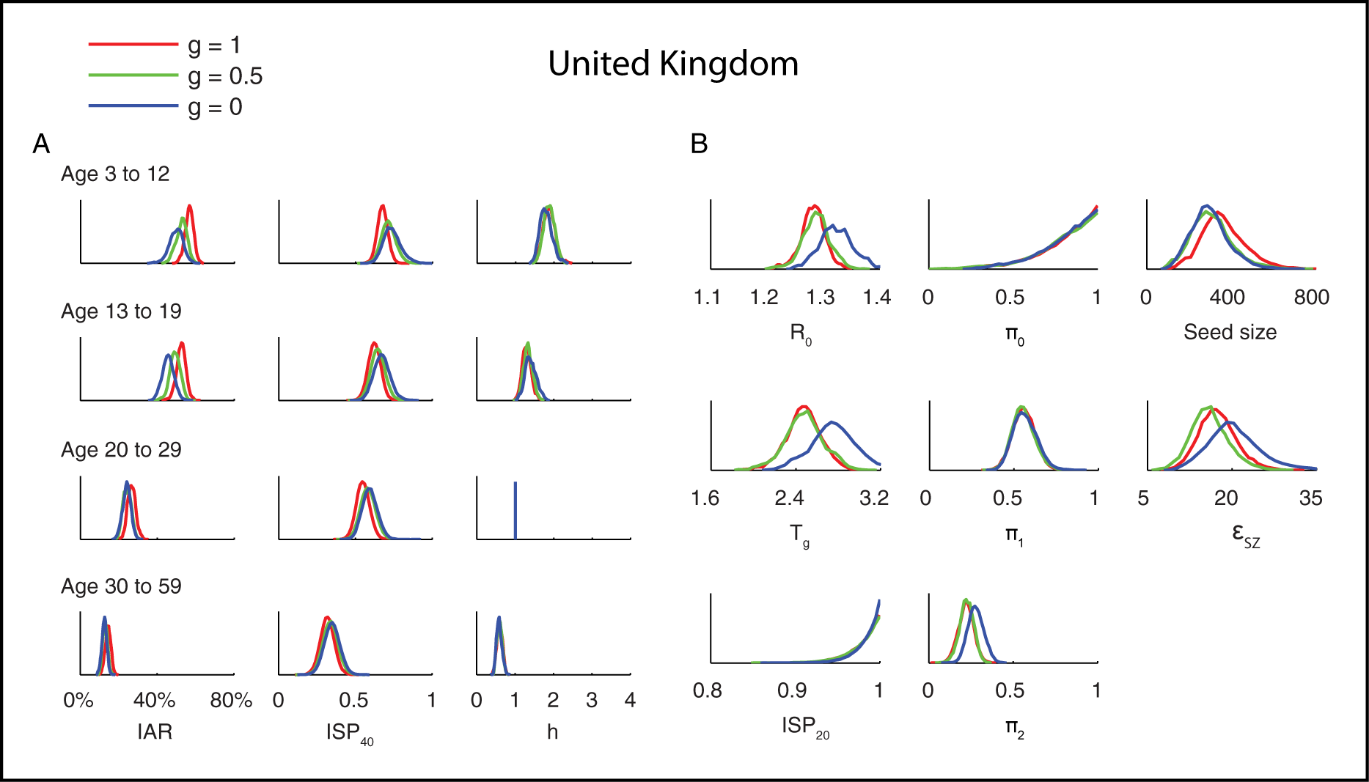
**

**Figure S12. Posterior distributions of parameters for different values of g with the United Kingdom POLYMOD contact matrix. A.** Age-dependent parameters including IARs (first column), *ISP*_40_ (second), and age-specific susceptibility (third). **B.** Other parameters including *R*(0), *T_g_,* *ISP*_20_, reduction in within-age-group mixing due to school closure (*π*_0_, *π*_1_, *π*_2_), seed size, and scaling factor for FOI from Shenzhen (*ε_SZ_*). **Higher g (i.e. preexisting MN titer conferred weaker protection) resulted in slightly higher IARs and lower *R*(0) and *T_g_*.**

# References

1. Keeling MJ, Rohani P (2008) Modeling infectious diseases in humans and animals. Princeton: Princeton University Press. xi, 366 p. p.

2. Wearing HJ, Rohani P, Keeling MJ (2005) Appropriate models for the management of infectious diseases. PLoS Med 2: e174.

3. Åke S (2007) A note on generation times in epidemic models. Mathematical Biosciences 208: 300-311.

4. Wu JT, Ho A, Ma ESK, Lee CK, Chu DKW, et al. (2011) Estimating Infection Attack Rates and Severity in Real Time during an Influenza Pandemic: Analysis of Serial Cross-Sectional Serologic Surveillance Data. PLoS Med 8: e1001103.

5. Mak GC, Choy PWW, Lee WY, Wong AH, Ng KC, et al. (2010) Sero-immunity and serologic response to pandemic influenza A (H1N1) 2009 virus in Hong Kong. Journal of Medical Virology 82: 1809-1815.

6. Veguilla V, Hancock K, Schiffer J, Gargiullo P, Lu X, et al. (2011) Sensitivity and Specificity of Serologic Assays for Detection of Human Infection with 2009 Pandemic H1N1 Virus in U.S. Populations. Journal of Clinical Microbiology 49: 2210-2215.

7. Cox CM, Goodin K, Fisher E, Dawood FS, Hamilton JJ, et al. (2011) Prevalence of 2009 pandemic influenza A (H1N1) virus antibodies, Tampa Bay Florida--November-December, 2009. PLoS ONE 6: e29301.

8. Baguelin M, Hoschler K, Stanford E, Waight P, Hardelid P, et al. (2011) Age-Specific Incidence of A/H1N1 2009 Influenza Infection in England from Sequential Antibody Prevalence Data Using Likelihood-Based Estimation. PLoS ONE 6: e17074.

9. Hardelid P, Andrews N, Hoschler K, Stanford E, Baguelin M, et al. (2010) Assessment of baseline age-specific antibody prevalence and incidence of infection to novel influenza A/H1N1. Health Technology Assessment 14: 115-192.

10. Miller E, Hoschler K, Hardelid P, Stanford E, Andrews N, et al. (2010) Incidence of 2009 pandemic influenza A H1N1 infection in England: a cross-sectional serological study. The Lancet 375: 1100-1108.

11. Reed C, Katz JM, Hancock K, Balish A, Fry AM, et al. (2012) Prevalence of Seropositivity to Pandemic Influenza A/H1N1 Virus in the United States following the 2009 Pandemic. PLoS ONE 7: e48187.

12. Nolan T MJSM, et al. (2010) Immunogenicity of a monovalent 2009 influenza a(h1n1) vaccine in infants and children: A randomized trial. JAMA 303: 37-46.

13. Dudareva S, Schweiger B, Thamm M, Höhle M, Stark K, et al. (2011) Prevalence of Antibodies to 2009 Pandemic Influenza A (H1N1) Virus in German Adult Population in Pre- and Post-Pandemic Period. PLoS ONE 6: e21340.

14. Bandaranayake D, Huang QS, Bissielo A, Wood T, Mackereth G, et al. (2010) Risk Factors and Immunity in a Nationally Representative Population following the 2009 Influenza A(H1N1) Pandemic. PLoS ONE 5: e13211.
